# Functional PET for mapping metabolic dynamics in Parkinson’s disease

**DOI:** 10.1038/s41598-025-28456-x

**Published:** 2025-11-18

**Authors:** Vanessa Heinecke, Lilly Machholz, Kenan Steidel, Lenna M. Rüsing, Falk K. Thiemig, Damiano Librizzi, Maya Beckersjürgen, Jennifer Fuchs, Markus Luster, Lars Timmermann, David Pedrosa, Marina C. Ruppert-Junck

**Affiliations:** 1 Neurology Department at Medical Faculty Marburg, Marburg University, 35043 Baldingerstraße, Marburg, Germany; 2 Clinic for Neurology, University Hospital of Marburg and Gießen, Marburg, Germany; 3 Donders Institute for Brain, Cognition and Behaviour, Centre of Expertise for Parkinson & Movement Disorders, Radboud University Medical Center, Nijmegen, 6525 EN The Netherlands; 4 Center for Mind, Brain and Behavior - CMBB, Universities Marburg and Gießen, Marburg, Germany; 5 Nuclear Medicine Department, Marburg University, Marburg, Germany

**Keywords:** Glucose dynamics, Metabolic variation, Seed-based metabolic connectivity, Neurocognitive networks, Cognitive symptoms, Non-motor symptoms, Imaging biomarkers, Computational biology and bioinformatics, Diseases, Neurology, Neuroscience

## Abstract

Static [^18^F]-FDG PET studies have shown characteristic metabolic patterns for Parkinson’s disease (PD). However static PET scans offer only a snapshot of synaptic activity, preventing insights into dynamics of glucose consumption on individual level. In this study, we apply a novel dynamic constant infusion PET protocol in combination with MRI for mapping metabolic dynamics in PD. Metabolic time series comparison revealed hypometabolism in bilateral clusters in the substantia nigra in PD patients compared to controls (p_FWE_ < 0.001). The temporal structure allowed us to depict metabolic networks covering the basal ganglia circuits and typical neural resting-state networks on individual level. A measure of time series variation showed a region-specific increase in time series variation in PD, which related to cognitive PD symptoms. The findings pinpoint the methodological capabilities of the acquisition protocol for evaluating metabolic dynamics and metabolic network activity on subject-level in PD. Our study lays a foundation for the application of molecular imaging with temporal resolution in context of neurodegenerative diseases. Such protocols can capture interregional metabolic changes on patient level, the applicability of which should be evaluated in prodromal stages.

**Supplementary Information:**

The online version contains supplementary material available at 10.1038/s41598-025-28456-x.

## Introduction

Parkinson’s disease (PD) is the neurodegenerative disease with the fastest growing prevalence worldwide^1^. PD is primarily considered a movement disorder, although the complex clinical manifestation, including non-motor symptoms, are not solely attributable to dopaminergic depletion in basal ganglia-cortex loops. Instead, network-level dysfunction is assumed to underly PD and specifically, more widespread aberrant interregional neural communication^2^. [^18^F]-fluorodeoxyglucose ([^18^F]-FDG) positron emission tomography (PET), as a direct correlate of neural activity indexing synaptic activity, has increasingly been utilised for brain network visualisation and holds significant potential as a network biomarker candidate^3^.

A stereotypic PD-related metabolic pattern with hypometabolism in occipito-parietal regions and hypermetabolism in the supplementary motor area and the putamina relative to healthy controls has been repeatedly observed^4^. In particular, it is well established that hypometabolism in the occipital cortex^5^ and regions covering the default-mode network relate to cognitive decline in PD^6^. Reliability of former results have been hampered by the static nature of classical PET acquisitions, providing only a snapshot per subject, so that only limited insights into metabolic dynamics and interregional communication at the individual level have been possible to date. Nonetheless, this information is crucial for uncovering network-level metabolic correlates of individual symptoms. Only recently, Villien et al. introduced a functional [^18^F]-FDG-PET (fPET) protocol leveraging constant tracer infusion and list-mode acquisition for information on glucose dynamics in the individual subject^7^. The technique offers a direct and quantitative measure of neuronal function^8^. In young healthy subjects, Voigt et al. found a strong association between cognition and metabolic network activity in fronto-parietal areas^9^. Irrespective of this, time series variation measures of the fMRI signal have been identified as key drivers of cognitive performance in aging^10^. However, metabolic networks and glucose dynamics at the subject-level remain underexplored within the context of neurodegenerative disorders despite their potential role for symptom expression.

**Fig. 1:**
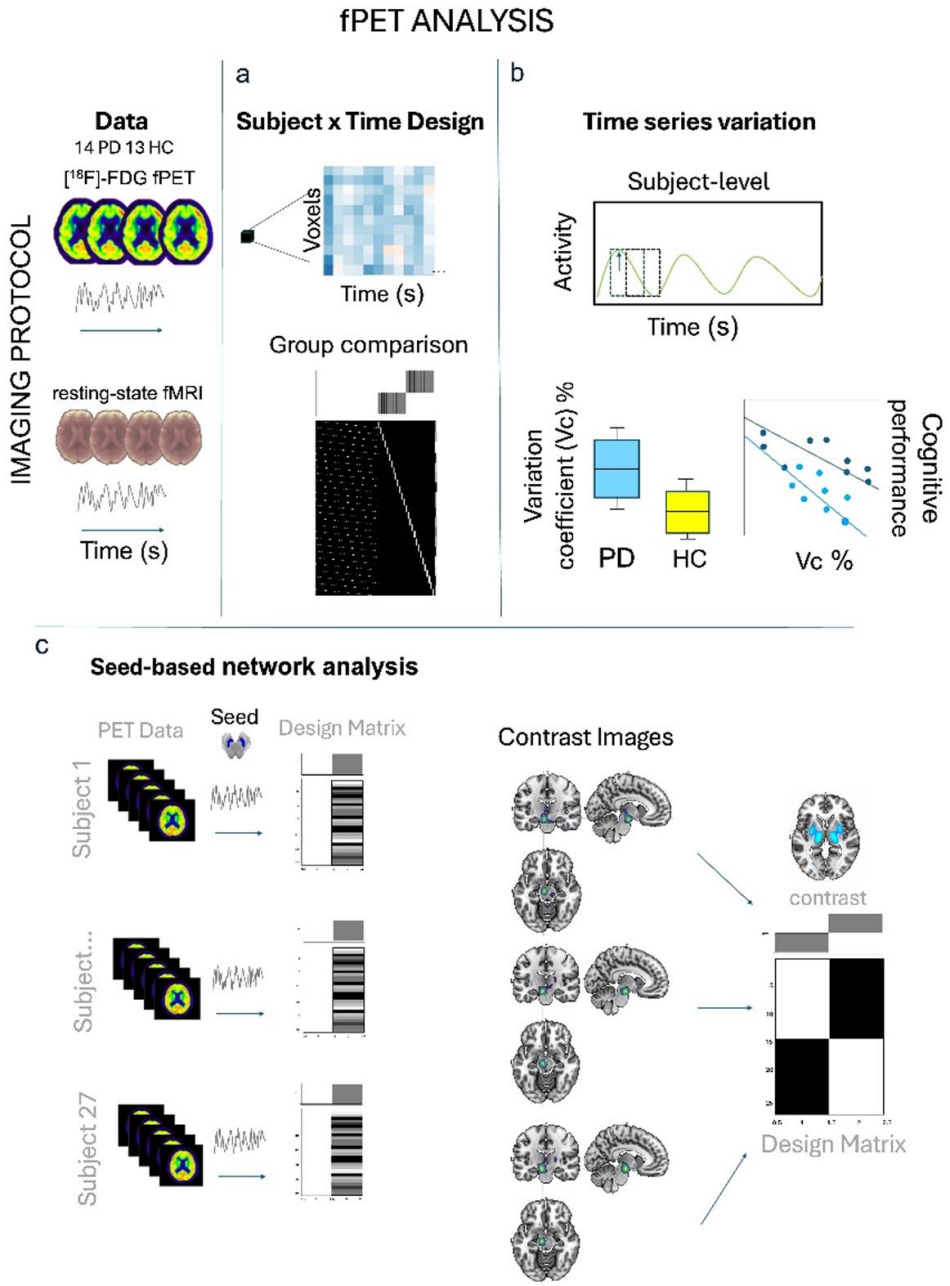
Schematic overview of the analysis pipeline enabled by the dynamic constant infusion fPET protocol. The temporal structure of our data provides several novel avenues in context of studying metabolic changes in neurodegenerative diseases: metabolic activity within the time series can be analysed in a subject x time design, providing detailed molecular information per subject (**a**), within-subject variation in metabolic activity can be studied and related to group level and behavioral variables (**b**), seed-based connectivity can be studied on individual level based on metabolic time series, directly compared to fMRI connectivity maps and compared between groups (c). VC = coefficient of variation, fPET = functional positron emission tomography, HC = healthy controls, PD = Parkinson’s disease.

To close this gap, we combined resting-state [^18^F]-FDG-fPET with fMRI acquisition to study glucose dynamics in PD. The combination of both modalities enabled us to analyse cross-modal relationships between glucose dynamics and hemodynamic networks. In addition, comparative networks at the subject level could be represented using fMRI as the current standard and fPET as a more direct measure of synaptic activity. We aimed to evaluate between-group differences in glucose metabolism based on time series data compared to healthy subjects, to establish a measure of glucose dynamics at the subject-level, and to develop a seed-based network approach for analysing metabolic time series data (Fig. 1). This approach offers new insights into how glucose dynamics contributes to individual PD symptoms and is related to network-level changes. The metabolic time series data allowed us to identify hypometabolic clusters in a comparatively small PD sample with a spatial overlap with the substantia nigra, which is unparalleled to date. Moreover, we report an increase in time series variation in fPET data associated with cognitive symptoms in PD and seed-based metabolic networks on subject-level based on constant infusion fPET data. These findings may lay the groundwork for developing novel network-based imaging markers for neurodegenerative diseases using molecular time series information.

## Results

### Study participants and clinical characterisation

A total of 14 persons with PD (PwPD) (63.4 ± 8.9 years, three females) and 13 healthy controls (59.5 ± 5.1 years, six females) underwent [^18^F]-FDG-fPET scanning. Cognitive screening tests did not reveal differences in cognitive function between the groups (Montreal Cognitive Assessment (MoCA): W = 118.5, *P* = 0.2). The average MDS-UPDRS-III score for PwPD without medication was 35.6 ± 17.6 points and the median Hoehn and Yahr stage was 2 [2.00–2.5], indicating mild to moderate severity of overall motor symptoms. According to MDS-UPDRS-III hemibody scores seven patients were left lateralised and seven right lateralised (refer to supplementary Table 1 for clinical and demographic information).

### Nigral hypometabolism and hypermetabolic activity in the motor cortex in PD

**Fig. 2:**
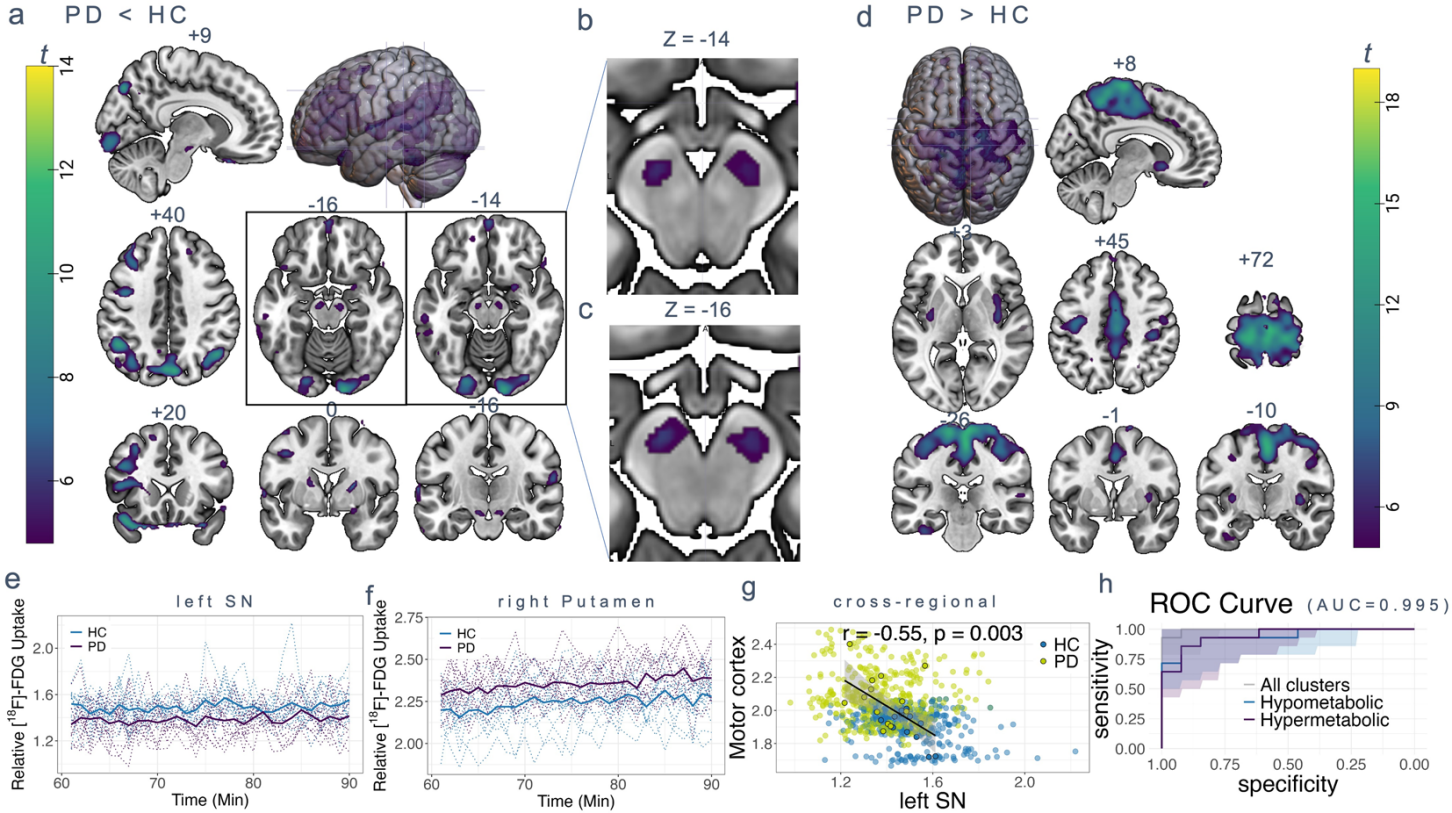
Hypometabolic and hypermetabolic clusters in PwPD relative to healthy controls based on metabolic time series comparison. SPM result of HC > PD visualised on MNI standard template. Sagittal, coronal and axial view of regions of hypometabolic (**a-c**) or hypermetabolic (**d**) activity obtained by voxel-wise group comparison of [^18^F]-FDG fPET scans from 13 healthy controls and 14 PD patients (*P* < 0.05 after FWE cluster level correction, extent threshold > 20 voxels). (**e-f**) Extracted individual and group mean time courses per cluster (MNI peak coordinates). (**g**) Regression plots for subcortical hypometabolism vs. cortical hypermetabolism (filled dots represent mean uptake per region). (**h**) Receiver operating characteristic curve illustrating the trade-off between sensitivity and specificity for group classification based on all clusters, hypometabolic or hypermetabolic clusters respectively. All images are shown in neurological display. PD = Parkinson’s disease, HC = healthy controls, SN = substantia nigra.

The voxel-wise group comparison of cerebral [^18^F]-FDG uptake time series using a flexible factorial design revealed subcortical and cortical regions with altered metabolic activity in PwPD compared to controls (*P* < 0.05 after family-wise error (FWE) rate correction). Regional hypometabolism was observed in the temporal, parietal, occipital and frontal lobes in PwPD (Fig. 2a). Parietal clusters included the bilateral angular gyri and precuneus (P_FWE_<0.0001, Supplementary Table 2). Subcortical hypometabolism was present in the basal ganglia: the bilateral substantia nigra pars compacta and reticulata (Fig. 2a-c), the putamina and the left caudate nucleus (P_FWE_<0.0001, Supplementary Table 2). The cerebellum, the right amygdala, the left insula and the dorsal part of the thalamus also showed relative hypometabolism in PwPD (P_FWE_<0.0001, Supplementary Table 2). Significant hypermetabolic regions in PwPD were observed in a large cluster spanning the supplementary motor area, the middle cingulate and the precuneus (Fig. 2d, P_FWE_<0.0001, Supplementary Table 2). The putamina, the orbitofrontal cortex, the medial occipital lobes, the fusiform gyrus and the cerebellum exhibited increased activity in PwPD (P_FWE_<0.0001). The right nucleus accumbens also showed relative hypermetabolism in PwPD (P_FWE_<0.0001, Supplementary Table 2).

Extracted relative [^18^F]-FDG uptake values from all clusters revealed contrast-specific patterns of signal time courses within hypometabolic clusters with a lower level of metabolic activity over time in PwPD (Fig. 2e) and hypermetabolic clusters characterised by a higher activity over time in PwPD (Fig. 2f). The corresponding time course for a longer interval from minute 40–90 can be found in the supplementary material (Supplementary Fig. S3). Contrasting the individual [^18^F]-FDG activity between hypometabolic clusters in the substantia nigra and hypermetabolic clusters in the motor cortex indicated that reduced subcortical activity is accompanied by corresponding increased cortical activity in PwPD (Fig. 2g). On the other hand, healthy controls’ interregional association was characterised by higher subcortical activity and lower cortical activity. Entering the extracted values into a group classification analysis yielded an area under the curve of 0.995 for a model that included all clusters with differences between groups, and 0.940 for hypometabolic or 0.945 for hypermetabolic clusters respectively (Fig. 2h). An additional analysis of cohen’s d revealed an effect size of 1.31 for the left and 1.24 for the right substantia nigra and confidence intervals of 0.04–0.17 for the left and 0.04–0.17 for normalised uptake values from the right substantia nigra. We compared motor lateralisation with the lateralisation of the most reduced nigral FDG uptake and found a corresponding association between most affected hemibody and stronger reduced contralateral nigral uptake in 9 out of 14 PD patients. In five patients, the left hemibody was clinically more affected but the strongest deficit also localised left-sided. The corresponding comparisons of static mean scans revealed no significant hypometabolism at the applied threshold and small clusters with hypermetabolism in the motor cortex at uncorrected p-level (not shown). Performing an ANCOVA per cluster with age as covariate underscored our findings apart from the right putamen cluster which did not surpass the threshold (*P* = 0.051). Additionally including MoCA scores, again the right putamen and left putamen did not surpass the threshold (*P* = 0.075, *P* = 0.08), but the remaining clusters still exhibited the reported group differences. ANCOVA analyses in the patients group revealed a significant effect of LEDD on metabolic activity in the substantia nigra clusters, which was independent of the variables age, disease duration and MoCA (RSN: *P* = 0.009, Eta = 0.701, LSN: *P* = 0.020, gEta = 0.620). In addition, there was a significant effect of MoCA in the left SN (*P* = 0.033, gEta = 0.558) and age in the right SN (*P* = 0.026, gEta = 0.591), which was independent of the other variables. A significant effect for age was also found in the hypermetabolic putamen cluster (*P* = 0.002, gEta = 0.828).

### Nigral metabolic and hemodynamic networks on subject level and group level

**Fig. 3:**
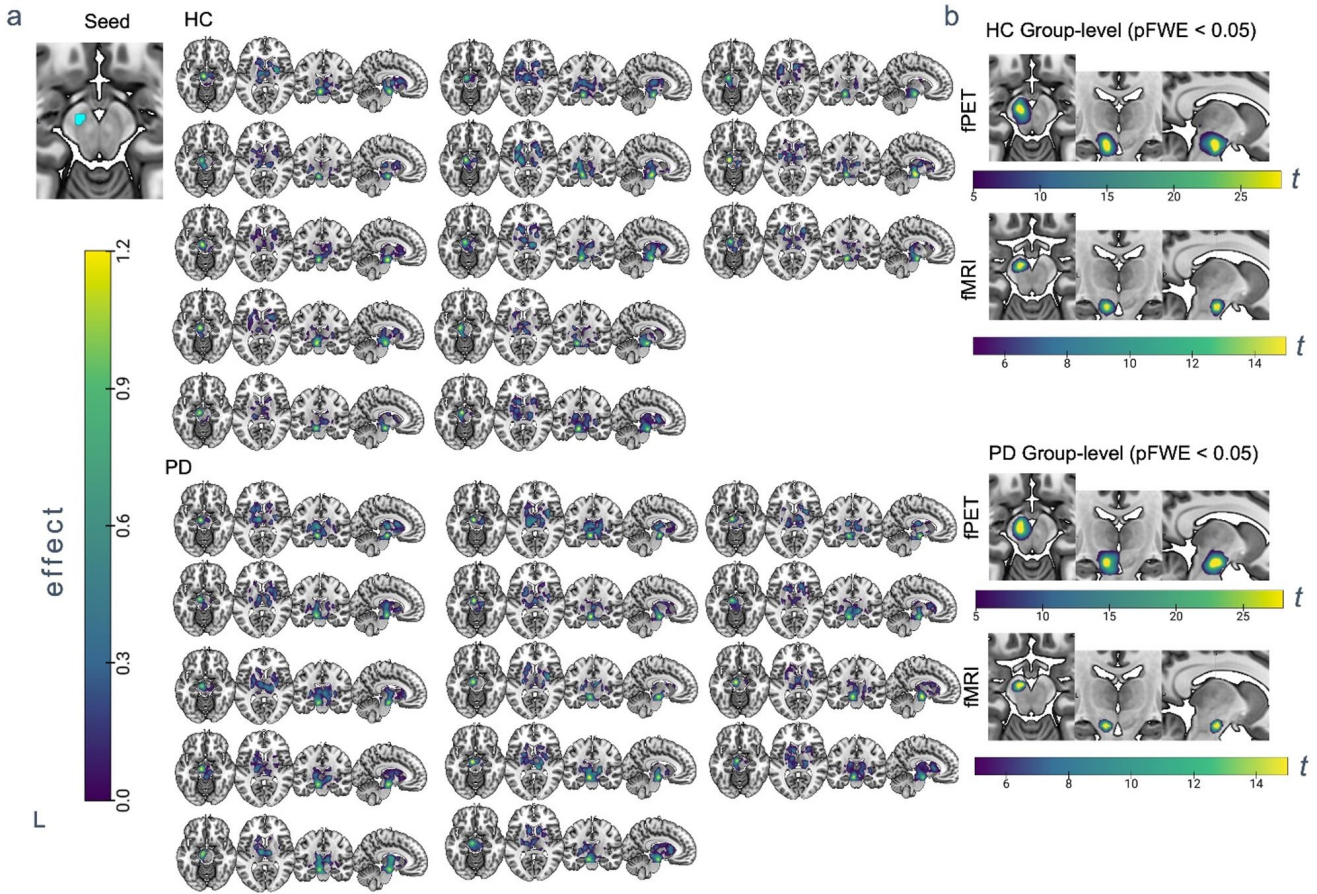
Seed-based nigral networks in PwPD and healthy controls on subject- and group-level. (**a**) SPM result of first-level single-subject regression analysis using the left substantia nigra as seed visualised on MNI standard template. Sagittal, coronal and axial view of regions of subject-level contrast images. Color bar represents subject-level effect in contrast images. Displayed slices are positioned at axial z = −14, + 8, coronal y= −14 and sagittal x= −9. (**b**) Group-level *t*-maps obtained by voxel-wise analysis of [^18^F]-FDG PET contrast images from 13 healthy controls or 14 PD patients (*P* < 0.05 after FWE cluster level correction). Color bar represents *t*-values of a voxel wise one-sample *t*-test of contrast images.

Seed-based metabolic connectivity analysis of the data-driven obtained substantia nigra clusters at the subject-level revealed the highest connectivity to nearby areas in the midbrain in all subjects for both sides (Fig. 3b fPET, Supplementary Fig. 1b), which remained significant at a threshold of *P* < 0.05 after FWE correction at cluster-level. At a more liberal threshold, additional clusters in the right and left putamen, right caudate nucleus, right pallidum and in the left ventrolateral thalamus, bilateral ventroposterior thalamus, left inferior lateral thalamus, left anterior pulvinar, medial pulvinar were obtained in healthy controls for the left substantia nigra seed (t > 2, uncorrected P-level). In PwPD, the midbrain cluster extended into thalamic regions including mediodorsal thalamus, left medial pulvinar, left inferior lateral thalamus, left ventrolateral thalamus at a more liberal threshold for the left substantia nigra seed (t > 2, uncorrected P-level). In addition, the left caudate, bilateral putamen and left pallidum were connected to the left nigral seed region in PwPD. Subject-level metabolic connectivity maps described consistently for all subjects the highest connectivity to nearby left midbrain regions (Fig. 3a). In all subjects, the left substantia nigra was connected to the left thalamus, left putamen and for some subjects also to the contralateral putamen or caudate nucleus. Similar results were observed for the right substantia nigra seed (Supplementary Fig. 1a). In order to compare these networks with a known reference, the same analysis was repeated with fMRI data. At the group level and with the same threshold (*P* < 0.05 FWE at cluster-level), this also revealed networks that were limited to the nearest surrounding areas (Fig. 3b fMRI).

### Alterations in region-wise glucose dynamics in PwPD relate to cognitive performance

**Fig. 4:**
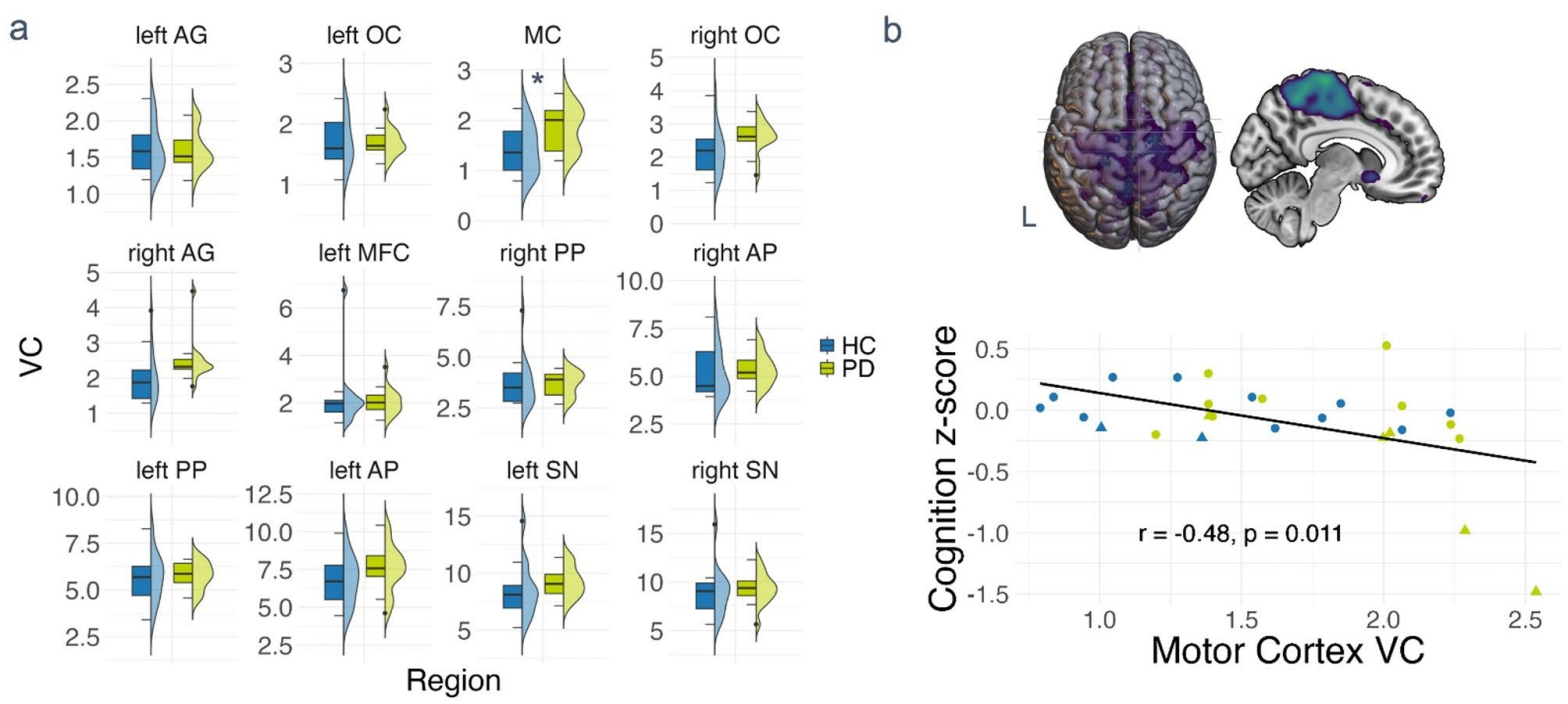
Metabolic time series variation in PwPD relative to healthy controls. Subject-level coefficients of variation from extracted metabolic time series in (**a**) regions with metabolic alterations in PwPD in the present cohort. Marked dots represent outliers with values > 1.5 times the inter-quartile distance. (**b**) shows a scatterplot relating regional coefficients of variation to cognitive performance measured by cognition z-scores. The subjects marked in rectanglular shape represent the subjects classified as MCI. All images are shown in neurological display. Abbreviations: HC = control subjects, PwPD = persons with Parkinson’s disease, SN = substantia nigra, OC = occipital cortex, AP = anterior putamen, MFC = mid frontal cortex, AG = angular gyrus, PP = posterior putamen, MC = motor cortex, VC = coefficient of variation.

A group comparison revealed a higher within-subject coefficient of variation within the cortical cluster covering the supplementary motor area, precuneus, and bilateral post- and precentral gyri (*P* = 0.02, Fig. 4a) in PwPD relative to healthy controls (effect size for the non-parametric Wilcoxon-test *r* = 0.43). Similar between-group differences were be obtained when calculating the coefficient of variation based on uptake values referenced to individual weight and FDG dosage (SUVr-values, *P* = 0.024). The subjects’ coefficient of variation of this region correlated significantly with cognitive performance evaluated by the screening tool MoCA (*r*= −0.44, *P* = 0.022) and cognitive z-scores, which included standardised performance scores from all cognitive domains (Fig. 4b, *r*=−0.48, *P* = 0.011). An independent analysis of atlas-based regions representing the key nodes of canonical resting-state networks showed that dynamic signal changes indicated by coefficient of variation occurred in the superior sensorimotor cortex in PwPD (*P* = 0.036, effect size: *r* = 0.38). Again, higher coefficients of variation in the superior sensorimotor cortex were associated with worse cognitive performance, measured by MoCA and cognitive z-scores (*r*=−0.42, *P* = 0.031, Supplementary Fig. 2b). A categorisation into groups according to cognitive performance based on common criteria revealed that five patients and two controls had mild cognitive impairment (see Supplementary Table 5 for detailed test results) and two out of these five exhibited the lowest cognition z-scores and highest coefficients of variation. The reported group differences in variation coefficients remained significant after controlling for age (*P* = 0.038, generalised Eta (gEta) = 0.167) and the effect of MoCA on variation within the motor cortex remained significant in the patient group after controlling for age, LEDD, and disease duration (*P* = 0.040, gEta = 0.531).

### Altered metabolic level and seed-based networks alongside cognitive decline in PD

**Fig. 5:**
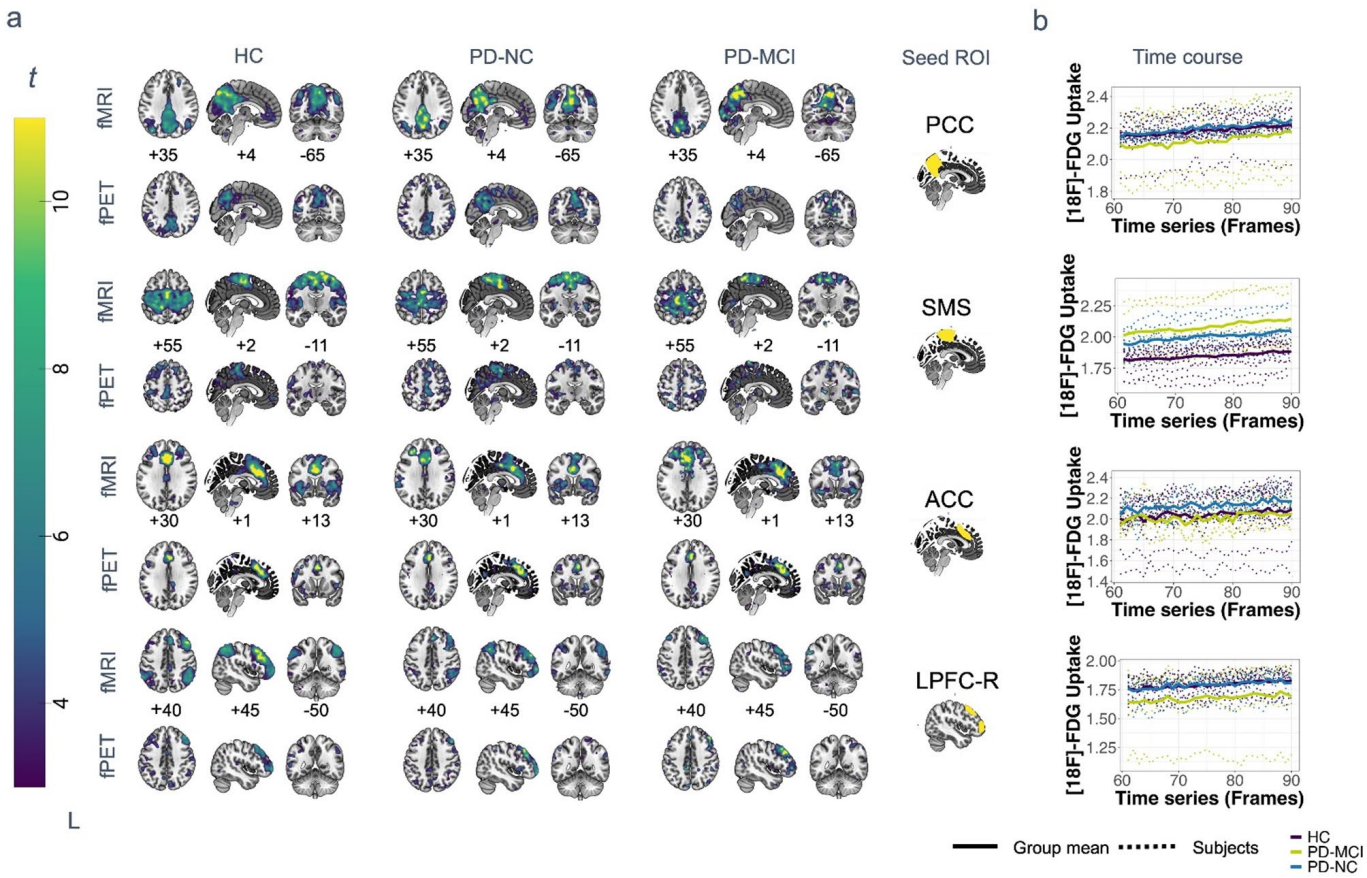
Seed-based metabolic and hemodynamic networks in PwPD with and without cognitive impairment and healthy controls. (**a**) Group-level metabolic (fPET) and hemodynamic (fMRI) seed-based networks obtained by voxel-wise one-sample *t*-tests with respective subject-level contrast images for *n* = 11 HC, *n* = 9 PD-NC (fMRI: *n* = 7) and *n* = 5 PD-MCI. (**b**) Extracted fPET time course of the corresponding seed region with subject-level time courses (dotted line) and mean time course per group (filled line). Colorbar indicates t-values between 3 and 11 for comparability of the modalities. All images are shown in neurological display. Abbreviations: HC = control subjects, PD = Parkinson’s disease, ACC = anterior cingulate cortex, LPFC-R = right lateral prefrontal cortex, PCC = precuneus cortex, SMS = superior sensorimotor cortex, MCI = mild cognitive impairment, NC = normal cognition, ROI = region of interest.

Seed-based metabolic and hemodynamic networks for four typical resting-state network nodes were evaluated on group level (*P* < 0.05 after FWE correction on cluster-level).The implemented seed-based approach for metabolic time series data revealed the typical default mode network structure in both independent imaging modalities in control subjects (Fig. 5a, supplementary Table 6). The obtained maps contained the posterior cingulate cortex, precuneus and inferior parietal cortex, which was only included on the left hemisphere in the fPET modality (Fig. 5a). In both modalities, less clusters were observed in PwPD and normal cognition and the least in PwPD and MCI (Fig. 5a). The extracted time series yielded the lowest [^18^F]-FDG uptake in the precuneal cortex and the highest [^18^F]-FDG uptake in PwPD and normal cognition and control subjects in between (Fig. 5b). By utilising a seed in the superior sensorimotor cortex, a typical cortical motor network was observed in all the groups in the fMRI modality (Fig. 5a). While PwPD with mild cognitive impairment showed a more posteriorly distributed network and loss of lateral clusters, patients with normal cognition exhibited only small deficits in the precentral gyrus in comparison to controls. Conversely, the fPET motor networks included more clusters in the frontal lobe and subcortical clusters, which were absent in the fMRI modality in all groups (Fig. 5a). The extracted time series revealed significant between-group differences (H = 9.1, *P* = 0.01) with the lowest [^18^F]-FDG uptake in the superior sensorimotor cortex in controls and a significantly higher [^18^F]-FDG uptake in PwPD with normal cognition in comparison to controls (*P* < 0.01 after Holm-Bonferroni correction) and the highest [^18^F]-FDG uptake in patients with MCI (Fig. 5b). Using the anterior cingulate cortex, the common structure of the salience network was observed in all groups in the fMRI modality (Fig. 5a). In contrast, fPET networks were more restricted to the seed. The extracted time course revealed the lowest activity over time in the PD group with cognitive impairment and the highest in PwPD with normal cognition (Fig. 5b). The right dorsolateral prefrontal cortex was connected to the inferior parietal cortex with similarly located clusters in both modalities, smaller clusters in fPET and less parietal clusters in the group of PwPD with MCI. The extracted time course showed a comparable activity in PwPD and healthy controls and lowest activity in patients with MCI over time (Fig. 5b). An analysis of signal stability in the resting-state network nodes revealed a good temporal signal-to-noise ratio (tSNR) in all subjects and no between-group differences in any node.

## Discussion

**Fig. 6:**
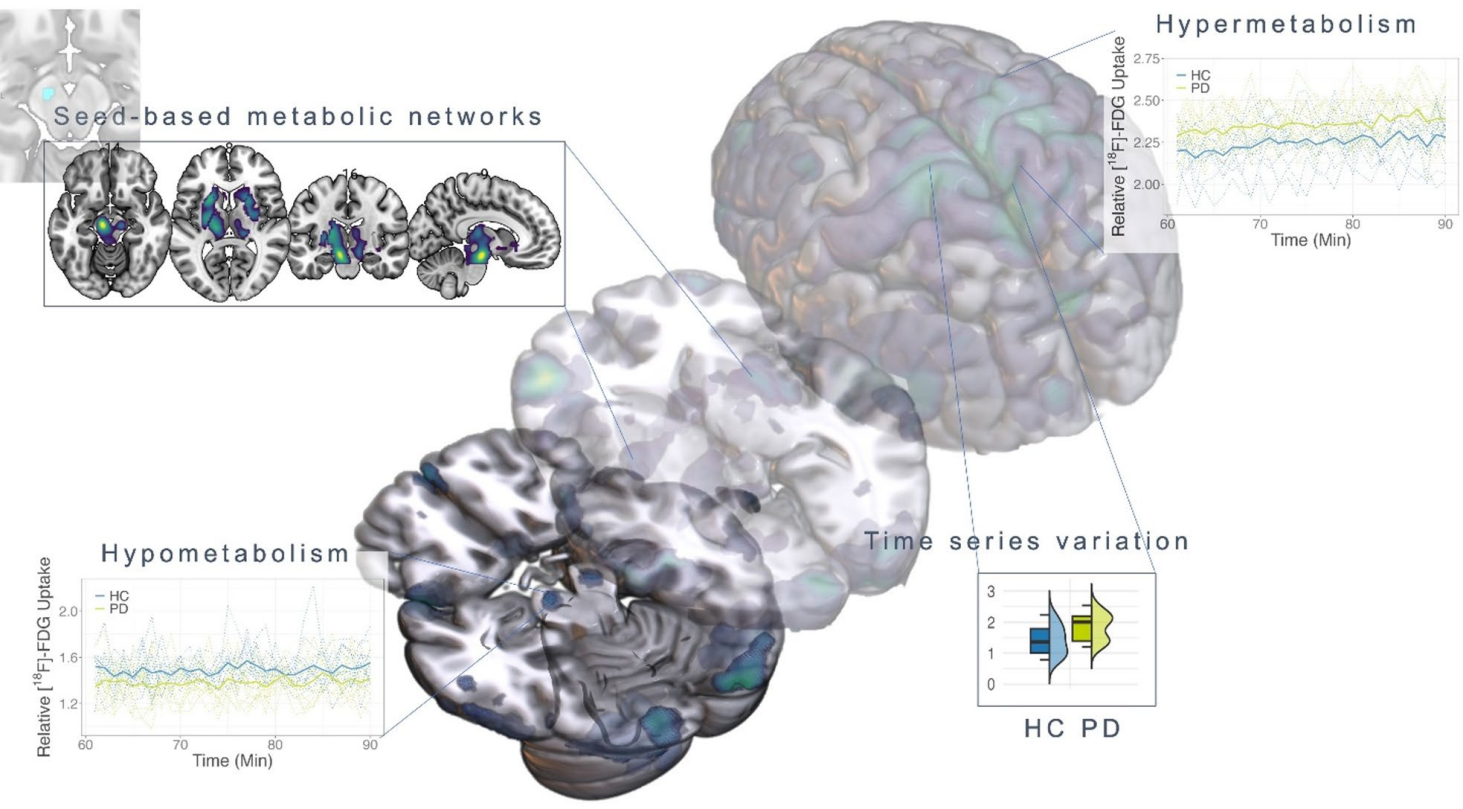
Schematic overview of metabolic features captured by the constant infusion fPET protocol in PD. Our study pinpoints three main effects on characterising PD-related metabolic changes based on this novel data structure. Firstly, we detected a typical pattern, including a bilateral hypometabolic cluster in the substantia nigra and hyperactivity in the motor cortex in a small PD sample. Second, symptom-related alterations in variations of glucose consumption on individual level could be captured. Lastly, the data structure enabled us to depict seed-based metabolic connectivity maps on subject-level, which revealed a metabolic network covering the basal ganglia for nigral seed regions.

Stereotypic metabolic patterns are promising candidates as circuit biomarkers for Parkinson’s disease. However, the methodological constraints of standard PET acquisition protocols, which provide only a static snapshot of glucose consumption, have hindered their routine application as interregional network markers on a subject-level. This study lays the groundwork for the application of a molecular imaging protocol with time series information in the context of neurodegeneration to overcome this hurdle. The key innovation of this protocol is the potential to derive metabolic time series reflecting glucose dynamics in the individual subject. We identified three PD-related metabolic changes based on this novel data structure. Firstly, we detected a typical pattern, including a bilateral hypometabolic cluster in the substantia nigra and hypermetabolic activity in the motor cortex in a small PD sample, which static mean scans could not capture. Secondly, symptom-related alterations in glucose dynamics at the individual level were identified and associated with distinct PD symptoms. Lastly, the data structure allowed us to depict metabolic connectivity maps on a subject-level, revealing subcortical basal-ganglia networks and typical cortical resting-state networks per subject based on metabolic time series information (Fig. 6).

The observed clusters with hypometabolism in PwPD in our study align with previously reported patterns, indicating occipito-parietal hypometabolism in PD based on static PET^5,11,12^. Subcortical hypometabolism in midbrain clusters encompassing parts of the substantia nigra revealed by voxel wise group comparison in a high-resolution static PET data set were first described by some of the authors of this work in 2020^12,13^ and also examined longitudinally as progression marker^14^. Another study reported similar findings with a region of interest-based approach in idiopathic PD as well as atypical parkinsonian syndromes and found lower metabolism in entities with worse nigrostriatal pathology^15^. We would like to point out that this part of the results is not based on a measure of dynamics but rather compares the level of glucose consumption between groups in the conventional manner, albeit with the novelty that time-resolved data acquired by means of constant tracer infusion is used. However, compared to the previous studies, the here reported subcortical clusters were even more restricted to the bilateral substantia nigra pars compacta and reticulata compared to previous studies as confirmed by the automated anatomical labeling atlas version 3 and the PD25 atlas. Given the small sample size and the absence of similar patterns in mean scans in our pilot study, it is plausible that the temporal resolution of the data with molecular time series information per subject allowed for the identification of more disease-related changes that correspond spatially with known spatial distribution of nigral cell loss. As LEDD is closely related to disease severity, the significant association between LEED and nigal metabolic activity after controling for the covariates age, disease duration and cognitive performance, supports that these metabolic changes are indeed disease-related. Additional multimodal imaging – such as neuromelanin-sensitive MRI and small animal PET studies in synucleinopathy models – may help corroborate the co-localisation with nigral pathology and dopaminergic cell loss^16^. Confirmation of a mechanistic association with nigral degeneration would underscore the translational potential of this imaging marker as a non-invasive tool for visualising nigral degeneration. There is preliminary evidence for nigral hypometabolism in patients with idiopathic rapid-eye movement sleep behavior disorder compared to matched controls by using an atlas-based approach with the median pons uptake value as the reference^17^.

The presented extracted time series from areas with changes in glucose consumption reveal important aspects for the following analyses, which actually relate to (1) changes over time and (2) interregional relationships that may not necessarily occur only in regions with altered glucose levels. Therefore, we first examined the variation in the signal in regions with changes in glucose consumption to investigate whether the variability of the signal in these regions also shows disease-relevant changes, but we also supplemented this analysis with atlas-based analyses.

Due to the static character of commonly applied PET protocols, the variation of glucose dynamics has only been addressed in one preprint in a pooled data set of younger and older subjects yet^18^. We report changes in the subject-level coefficient of variation in the data-driven motor cortex cluster as well as in an atlas-based superior sensorimotor region in PwPD with a moderate effect size. The level of variation within the time series was associated with cognition in our cohort with patients presenting with MCI showing the highest coefficients of variation in the superior sensorimotor cortex accompanied by a lower level of metabolic activity. Since the tSNR values did not differ between the groups, it is unlikely that these differences in metabolic activity over time were driven by noise. Only a few studies have examined comparable measures of the blood oxygenation level dependent (BOLD) signal variability. One study using a working memory task found age-related increases in BOLD variability in older adults, particularly in the left and right precentral gyrus^10^. In individuals aged ~ 20–66 years, greater variability was associated with poor performance both during and outside the scanner. Our data suggest a similar association between the superior sensorimotor area, including pre-motor areas with cognitive impairment in PD based on a direct measure of neural activity. Another study has reported atrophy in the precentral gyrus and supplementary motor area in PD with MCI, which was accompanied by hypermetabolic changes in PD with dementia in a longitudinal design^19^. Together both findings might be indicative of adaptive processes that result from atrophy in this region, which could not be analysed in the current data set due to a rather short structural MRI protocol. These findings should be analysed in longitudinal studies with larger sample sizes to examine the potential of these metabolic measures for detecting the progression of early cognitive dysfunction. In the mentioned preprint, higher glucose dynamics based on standard deviation, was associated with better cognitive performance, but more pointing towards an importance of regions of the salience network. The motor network, by contrast, was related to age effects in their study, showing reduced variability at higher age^18^. Nevertheless, these results do not contradict the current study insofar as a different measure (no relative measure) was used and no neurodegenerative patients were considered.

The temporal resolution also enabled us to investigate for the first time the relationship between metabolic time series in two regions at the subject level in a neurodegenerative disease. This resulted in the first connectivity measure based on metabolic time series, which was examined here in the context of a disease. The implementation of seed-based analysis for dynamic fPET data for the nigro-striatal system enabled the description of subcortical metabolic networks on subject-level. The nigral seed’s time course exhibited a plausible correlation with striatal and thalamic regions in all subjects often with continuous clusters to thalamic regions that appear like a continuous cluster between the midbrain and the thalamus. Two interesting aspects should be mentioned at this point. Firstly, a very recent study has provided evidence for a direct dopaminergic connection between the substantia nigra pars compacta and the thalamus in young healthy subjects by using multi-shell high-angular resolution diffusion MRI^20^. Second, the observed thalamic regions were among the most prominent regions within the a disease-specific metabolic network revealed by independent component analysis in this data set^21^: it is unknown how the here reported hypometabolic clusters, located ventrally to the brain stem part of the metabolic motor network, relate to the observed hypersynchronous motor network. If both findings are reflective of thalamic disinhibition in PwPD, is rather speculative and needs to be clarified in additional studies, incorporating larger sample sizes. Additionally, multimodal studies incorporating protocols optimised for small fibre tracking could reveal important insights into structural-metabolic correlates in neurodegeneration like recently reported in young healthy subjects^22^.

The implementation of seed-based analysis for dynamic fPET data enabled the identification of canonical resting-state networks based on metabolic time series. At the applied threshold, the highest spatial similarity between fPET and fMRI was observed in the DMN across the HC and PD-NC groups (dice coefficient: 0,17 in HC and 0,24 in PD-NC), whereas a low correspondence was observed in PD-MCI (0.07). The seed-based analyses presented here can be only compared to previous studies that applied static PET to PD cohorts. In accordance with Sala et al., fewer clusters were observed in the prefrontal cortex in PD, with no clusters found in PwPD and MCI^23^. While the motor network identified using our superior sensorimotor cortex seed extended into lateral frontal and subcortical areas, such as the thalamus, in the PET modality, the fMRI network was confined to the pre- and postcentral gyri and supplementary motor area. Although the representation of the networks at the group level is of course still highly dependent on the group size, the results demonstrate that it is possible to use this technique to identify resting networks of comparable spatial extent through seed-based methods based on metabolic time series. The rationale behind identifying metabolic networks with close spatial similarity to canonical resting-state networks is partly based on fundamental science: the underlying signal for functional connectivity is seen as a rather indirect measure of neural activity. To date, resting-state networks have not been able to establish themselves as a reproducible marker. Our findings indicate that the fPET technique makes it possible to visualise a metabolic component of these known resting networks using a direct indicator of neural activity with temporal resolution. This could contribute to fMRI or, in the future, a combination of both resting-state modalities helping to establish a network marker that is more interpretable and robust thanks to the multimodal combination (also with other techniques such as tractography and dopaminergic imaging). In particular, the anatomical basis of fPET-based networks should be investigated in future studies, as initial evidence from covariance studies based on static PET shows a high degree of correlation with short- and long-range connections, which could be even higher when actual metabolic time series are used^24^.

Although our study provides important initial insights into metabolic time series in neurodegeneration, it has some limitations. Firstly, the study has a relatively small sample size, which was limited by the Federal Bureau for Radiation Protection and the ethics committee to answer the question at minimal possible risk. We acknowledge that the relatively small sample size reduces the power of our analyses, increasing the risk of type II errors and contributing to unstable or overestimated effect size estimates. Especially the analysis of subgroups with different cognition levels, are highly explorative and should be validated in larger samples. Secondly, we applied strict inclusion criteria to ensure that patients tolerate an off-phase longer than 12 h, which limits the generalisability of our findings to other disease stages.

In addition, our multimodal acquisition protocol is afflicted with some restrictions. As both imaging procedure were performed consecutively in different scanners at different day times, it may be of question whether both represent the same resting-state of brain function. However, several requirements were undertaken to guarantee comparability: both procedures were performed in rooms with dimmed light under standardised conditions and standardised instructions were utilised like in comparable studies^12^. Follow-up studies could investigate more direct associations between glucose dynamics and fMRI signals in a hybrid setting which was not possible using consecutive imaging.

Our resting-state network analyses revealed common large-scale networks with comparable spatial distribution in both modalities. However, it is important to acknowledge that fPET-based networks are still derived based on a lower temporal resolution compared to fMRI. Nevertheless, it is unlikely that the slow dynamics of neurometabolic coupling can be better resolved in seconds^8^. According to previous studies, task-based fPET can reliably address stimuli induced changes in the minute time scale^7^, particularly in 2–5 min lasting tasks, and shorter time frames are still afflicted with an insufficient SNR^25^. With 1-minute time frames, our reconstructed images have a temporal resolution that is within the typical resting-state frequency band used in resting-state fMRI studies (0.01–0.1 Hz), which has been applied in resting-state studies in PD and is able to detect symptom-related changes in functional connectivity in motor and cognitive networks. Accordingly, this time scale represents a promising scale to identify similar cross-regional correlations in slow metabolic fluctuations implicated in PD symptoms (see Fig. 5). There is currently still considerable variability in the application of subregional metabolic interactions using FDG-PET, known as metabolic connectivity. Volpi et al. provide strong evidence that technical variability in fPET-MC studies — in particular the choice of model parameters, metrics, time windows and analysis approach — can strongly influence connectivity results, and that the across-subjects covariance frequently used in the context of PD to date is characterised by interindividual differences and technical aspects rather than actual connections between regions^26^. Our work therefore makes an initial contribution to examining actual subject-level metabolic connectivity by collecting time series data based on fPET. In addition, also the choice of measure for time series variability may depend on the individual metrics chosen. While another preprint used the standard deviation^18^, we decided to include the coefficient of variation as it is relative measure with reference to the individual mean signal and dimensionless, enabling comparisons across regions and groups. Future studies could also consider more robust (interquartile range) or model-based metrices.

Due to a low activity in initial frames, only the last 30 frames with a duration of 1 min per frame were retained in the presented analyses and it needs to be considered that preprocessing pipelines for constant infusion fPET are not yet as standardised as fMRI processing pipelines including denoising and intensity normalisation^8^. Nevertheless, we chose the minimum possible reconstruction interval possible on our system (1-minute time intervals) and retained an analysis period comparable to previous fPET studies^7,27^. Of note, the kinetics underlying this protocol, which is fundamentally different from static bolus imaging in terms of tracer application, has been described in previous studies and simulations suggest that this technique is less sensitive to interindividual differences in physiological parameters^7,28^. Additionally, given the long acquisition time, there might be an effect of k4 (dephosphorylation) on glucose fluctuations, which is likely rather small and has been neglected in most fPET studies up to date. Future studies should elaborate a standardized technique for estimating k4 to incorporate this uncertainty into the assumptions.

Finally, mapping small nuclei in subcortical areas is always subject to uncertainty. Therefore, all available atlases, including Parkinson’s-specific atlases, were used to ensure that spatial mapping was as accurate as possible. Calculation of ROI-based measures, like the applied measure for time series variation, is dramatically dependent on ROI definition. Therefore, data-driven ROIs as well as atlas-based approaches were performed to validate the findings. additional ROI-based analyses depend on ROIs derived from the Human Connectome Project^29^. As described in Yaeger at al^30^., it needs to be considered that these ROIs were derived from young adults and the transferability to elderly subjects needs to be handled with caution^30,31^. As shown in many multimodal neuroimaging studies, functional network alterations can be co-localized with atrophy. In the current data set, we could not look at grey matter changes or cortical thickness as the anatomical MRI protocol was explicitly designed as a very brief and short scan for registration purposes to spare patients a longer lying time in the scanner in view of the extensive imaging protocol. Since functional changes in fMRI and atrophy are expressions of the same degenerative processes – only visible in different stages or modalities – atrophy analysis does not necessarily provide an additional value. In our opinion, the applied protocol is still in an optimisation phase. However, it has enormous potential in the field of biomarkers for neurodegenerative diseases, particularly for hybrid imaging techniques. We therefore propose standardising infusion (infusion rate, bolus + constant infusion) and recording protocols (acquisition time, reconstruction, number of iterations, matrix size) across centres and conducting studies with larger sample sizes based on joint consent. In addition, the evaluation pipelines should be standardised, particularly with regard to unclear issues such as autocorrelation and residual post-normalisation drifts and denoising steps.

## Conclusion

In conclusion, the results of this study support the use of the constant infusion fPET protocol in context of neurodegenerative diseases and demonstrate its ability to detect subcortical metabolic alterations in PD, unidentified by corresponding averaged mean scans. The findings are consistent with patterns obtained using static protocols and validate our previous findings regarding midbrain hypometabolism in an independent, non-high-resolution small data set. Our study provides first insights into subject-level glucose dynamics and network connectivity based on metabolic time series information in a neurodegenerative disease.

## Methods

### Study participants and data collection

The study received approval from the local ethics committee of the medical faculty of the Philipps-University of Marburg (146/19). Authorization for radiation exposure was obtained by the Federal Office for Radiation Protection. The study was carried out in adherence to the principles outlined in the Declaration of Helsinki and participants declared their written informed consent before participating.

A total of 14 healthy controls (HC) and 15 Parkinson’s disease (PD) subjects were recruited, of which 13 HC and 14 PD patients provided [^18^F]-FDG-PET data. PD subjects were recruited through the central study coordination of the Department of Neurology at the University Hospital of Marburg in Germany. HC subjects were recruited through advertisements. Inclusion criteria: german speaking, older than 50 years old, three to eight years of illness, Hoehn & Yahr (H&Y) stage 1–2.5 in motoric OFF-state, no therapeutic changes within three months. Patients needed to be able to endure a medication break of 12 h of non-retard and 72 h of retarded PD medication. Exclusion criteria: structural cerebral damage (e.g. vascular events, tumors), severe depression and motor complications, signs of dementia, safety concerns about MRI scanning like pacemaker, artificial heart valves, metal in the body (e.g. total endoprostheses) and claustrophobia, pregnancy and a blood glucose > 180 mg/dl at the time of PET examination.

### Clinical assessment

Motor severity was tested according to part III of the Movement Disorder Society Unified Parkinson`s disease rating scale (MDS-UPDRS-III) in ON and OFF-state^32^. The levodopa equivalent daily dose (LEDD) was determined using established criteria^33^. Motor lateralisation was calculated based on right and left body scores obtained from UPDRS-III off hemibody items. All subjects underwent a cognitive test battery including Montreal Cognitive Assessment (MoCA), revised Wechsler Memory Scale (WMS-R), Parkinson Neuropsychometric Dementia Asessment (PANDA)^34^, and Regensburg Word Fluency test (RWT).

The categorisation of all our subjects into mild cognitive impairment (MCI) and normal cognition (NC) was carried out according to Movement Disorder Society Level II criteria^35^. MCI was diagnosed in PD patients, when a difference of >/= 1.5*standard deviation was observed in relation to age-matched norm means in at least two cognitive test results regardless of domain affiliation.

### Resting-state [^18^F]-FDG-PET and fMRI acquisition

The dynamic [18 F]-FDG-PET scans were acquired on a SIEMENS Biograph 6 Scanner (Siemens, Germany) at the Department of Nuclear Medicine at the University Hospital of Marburg, Germany. Measurements of all subjects were carried out in OFF-state after overnight fasting and testing of blood sugar levels under standardised conditions. An average, 199.3 ± 5.3 MBq of [18 F]-FDG were infused via i.v. injection continuously using a perfusor (Braun, Germany) at a rate of 0.01 ml/s. During PET-scanning, we followed the same instructions as in the rsfMRI acquisition: patients were asked to keep their eyes open and do not think about anything in particular.

Following a low-dose CT transmission scan, PET data were acquired in list-mode for 90 min and saved for offline reconstruction as described in Villien et al.^7^. Resulting events were corrected for attenuation and sinograms were reconstructed into 90 1-minute frames with a matrix size of 168 × 168 (voxel size: 4.07 × 4.07 × 1.5 mm) using the 3D Ordered Subset Expectation Maximization (OSEM) algorithm implemented in the software Syngo (Siemens). Reconstructed frames were smoothed with a 5 mm Gaussian kernel, exported as DICOM files and subsequently converted into 4D NifTi file per subject using the dicom2niix tool in MRICroGL.

MRI scanning was performed on a Trio Tim Syngo 3 Tesla MR-scanner (Siemens, Erlangen) at the Department for Psychiatry and Psychotherapy of the University Hospital of Marburg, Germany. Participants underwent structural MRI with the following parameters: repetition time (TR): 1900 ms, echo time (TE): 2.5 ms, voxel size: 1.0 × 1.0 × 1.0 mm^3^. For fMRI measurements, subjects were instructed to keep their eyes open and to avoid thinking about anything in particular. The eye area was checked by camera throughout the measurement. The 8-minute lasting multiband echo-planar imaging sequence^36^ was characterised by the following parameters: 490 time points, TR 1040 ms, TE 30.0 ms, 3 × 3 × 3 mm^3^ voxel size and 48 slices. DICOM files were converted into NifTi files using the dicom2niix tool in MRICroGL.

The detailed overview about further preprocessing and analysis of the data is described in Ruppert-Junck et al. 2024^21^ and corresponding scripts are available on our Github repository: https://github.com/ruppertm/fPETDynamics.git.

### Neuroimaging data analysis

#### Voxel-wise group comparison in a subject-and-time design

A flexible factorial design in SPM12 was applied to evaluate voxel-wise differences in metabolic activity within the time series between healthy controls and PD patients, considering the last 30 frames of spatially normalised scans per subject. Two main effects were created: the first with factor number 1 (Subject) and the second with factor number 2 (Time). Global normalisation was conducted using ANCOVA with reference to the global mean. Global mean values did not differ between healthy subjects and PD patients (W = 120, *P* = 0.169). Additionally, a grey matter mask in MNI-space was applied to restrict the analysis to grey matter regions (ICBM 2009c non-linear symmetric, FSL). Results were considered statistically significant if *p* < 0.05 after family-wise error (FWE) rate correction at cluster-level and surpassed a minimum cluster size of 20 voxels. Anatomical labelling done by using the AAL v3 Atlas. Activity values per region were extracted using the MarsBaR toolbox in SPM12 and global mean-normalised values were plotted in R. Mean normalised uptake values per contrast were entered into a logistic regression and utilised to derive receiver operating characteristic curves per contrast or for all clusters. Cohen’s d was calculated as an additional measure of effect size.

#### Functional and metabolic connectivity analyses

The fMRI data set was preprocessed according to standardised procedures as described recently^21^. Seed-based resting-state functional connectivity analysis was performed with the default weighted general linear model using the CONN toolbox^29^. Anatomical labelling was performed by using the Harvard-Oxford atlas as implemented in CONN. We performed seed-based analyses with the observed hypometabolic midbrain cluster (left SN, right SN) to unravel the corresponding subcortical network and performed atlas-based analyses with four common resting-state network seeds which have been implicated in PD cognitive symptoms^37,38^. As a quantitative measure for cross-modal correspondence, the dice coefficient of similarity was applied. Results were considered statistically significant if *P* < 0.05 after FWE correction at cluster-level. As a measure of temporal signal stability, we calculated the temporal signal-to-noise ratio (tSNR) to assess the quality of time series data. The tSNR per ROI was calculated based on the following formula: tSNR = $\:\frac{{\mu\:}_{s}}{{\sigma\:}_{s}}$ with the mean signal over time divided by the standard deviation over time.

#### Metabolic connectivity within the striato-nigro-thalamic system

Seed-based metabolic connectivity analyses were performed using scripts relying on SPM12 in Matlab v23a which can be found on our Github Repository (https://github.com/ruppertm/fPETDynamics.git). Mean uptake time series were extracted from substantia nigra clusters per subject from global-mean corrected scans and utilised as covariate of interest in a voxel-wise regression analysis per subject. The obtained *t*-maps per subject were transformed into z-maps. All individuals’ first-level contrast images were entered into a one sample *t*-test for visualisation on group level and into second-level group comparison evaluating voxel-wise differences in metabolic connectivity. A masked analyses was performed focused on the striato-nigro-thalamic network by using as mask created with the corresponding regions from AALv1 and TD atlas. Subject-level contrast images were visualised with effects between 0.2 and 1.2 and group results presented at *P* < 0.05 after FWE correction.

#### Calculation of measures of glucose dynamics –coefficient of variation

As a measure of variation within the time series per subject, the coefficient of variation per region was calculated per region according to the following formular:

$$\:\text{V}\text{c}\:\left({\%}\right)=\frac{\sigma\:}{{\overline{x}}}\text{*}100$$

The coefficient of variation was calculated based on global mean normalised uptake values as well as standardized uptake values with reference to individual weight and FDG dosage (SUVr).

Differences between the groups were derived by permutation tests (https://github.com/ruppertm/fPETDynamics.git). The association to clinical variables (UPDRS-III, MoCa, cognitive z-scores) was analysed by linear regression and correlation analyses in R.

Network ROIs were defined via independent component analysis of Human Connectome Project data (*N* = 497)^29^. Network seeds are listed with x, y, z coordinates for the centroid of each seed.

### Statistical analysis of clinical data

Statistical analysis of clinical data were performed on demographic, behavioral and clinical data using R (RRID: SCR_001905)^39^. Group comparison were performed with two-sided Welch’s t-test or (*Mann-Whitney U test*) based on the results of Shapiro-Wilk test of normality. Multiple group comparisons were performed by using Kruskal-Wallis test and pairwise Wilcoxon tests with Bonferroni-Holm correction for multiple comparison.

## Supplementary Information

Below is the link to the electronic supplementary material.

Supplementary Material 1

## Data Availability

The data that support the findings of this study are available from the corresponding authors author upon reasonable request.
