# Supplementary material for: Functional PET for mapping metabolic dynamics in Parkinson’s disease
Source: Sci Rep. 2025 Nov 18;15:40334. doi: 10.1038/s41598-025-28456-x (PMC12627653; doi:10.1038/s41598-025-28456-x)
Supplement: Supplementary file 1 — Supplementary Material 1 [file 41598_2025_28456_MOESM1_ESM.docx]

**Supplementary material**

**Functional PET for mapping metabolic dynamics in Parkinson’s disease**

Vanessa Heinecke^1^, Lilly Machholz^1^, Kenan Steidel^1,2,3^, Lenna M. Rüsing^1,4^, Falk K. Thiemig^1,4^, Damiano Librizzi^5^, Maya Beckersjürgen^1^, Jennifer Fuchs^1^, Markus Luster^5^, Lars Timmermann^1,2,4^, David Pedrosa^1,2,4,#^ and Marina C. Ruppert-Junck^1,2,4,#,*^

1 Neurology Department at Medical Faculty Marburg, Philipps-University Marburg, Marburg, Germany

2 Clinic for Neurology, University Hospital of Marburg and Gießen, Marburg, Germany

3 Donders Institute for Brain, Cognition and Behaviour, Radboud University Medical Center, Centre of Expertise for Parkinson & Movement Disorders, 6525 EN Nijmegen, The Netherlands

4 Center for Mind, Brain and Behavior - CMBB, Universities Marburg and Gießen, Marburg, Germany

5 Nuclear Medicine Department, Philipps-University Marburg, Marburg, Germany

| Variable | HC (n = 13) | PD (n=14) | Test Statistics | Statistics | p-value |
| --- | --- | --- | --- | --- | --- |
| Age | 59.54±5.13 | 63.43±8.92 | t | -1.40 | 0.18 |
| Sex (m/f) (n) | 7/6 | 11/3 | Odds ratio | 0.33 | 0.237 |
| PANDA Sum | 24.92±4.54 | 23.43±6.16 | w | 100.50 | 0.66 |
| MoCa Sum | 28.00±1.91 | 25.86±4.70 | w | 118.50 | 0.19 |
| BIS11 Sum | 56.31±6.76 | 59.71±10.37 | w | 73.50 | 0.41 |
| UPDRS-III | - | 35.64±17.54 | - | - | - |
| LEDD (mg) | - | 668.88±358.25 | - | - | - |
| Hoehn & Yahr | - | 2 [2.0-2.5] | - | - | - |
| Lateralisation (MDS-UPDRS III) (n/n) | - | 7/7 | - | - | - |

**Supplementary Table 1: Demographic, clinical and behavioral data of subjects who underwent fPET scanning.** Abbreviations BIS-11 = Barret Impulsiveness Scale-11, HC = Healthy control, LEDD = Levodopa equivalent daily dose, MDS-UPDRS-III – Movement Disorder Society Unified Parkinson’s disease Rating scale, part III, MoCA = Montreal Cognitive Assessment, OR = Odds ratio, PD = Parkinson’s disease, PANDA = Parkinson Neuropsychometric Dementia Assessment. All results are reported as mean±sd apart from Hoehn and Yahr scale, which is reported as median and interquartile distance.

| contrast | region | MNI-coordinates (x y z) | t-value | cluster size  (number of voxels) | p_FWE_ |
| --- | --- | --- | --- | --- | --- |
| HC>PD | Temporal pole sup L, OFC post L | -36 24 -28 | 13.41 | 386 | <0.0001 |
|  | Angular L, Supramarginal L, Postcentral L, Temporal mid L, Temporal sup L, Parietal inf L  Precuneus L,  Precuneus R | -52 -58 24 | 12.39 | 4865 | <0.0001 |
|  | Frontal mid L, Frontal sup L, Frontal inf tri L, Frontal inf oper L, Insula L, Caudate L | -34 48 02 | 12.17 | 3135 | <0.0001 |
|  | Angular R, Temporal mid R, Occipital mid R | 46 -62 36 | 11.81 | 1764 | <0.0001 |
|  | Calcarine L, Occipital mid L, Lingual L, Occipital inf L, Cereb Crus 1 | -14 -98 -04 | 10.67 | 2017 | <0.0001 |
|  | Lingual R, Occipital inf R, Calcarine R | 14 -96 -16 | 10.63 | 1124 | <0.0001 |
|  | Occipital sup L, Cuneus L | -14 -98 26 | 9.32 | 65 | <0.0001 |
|  | Precentral L, Frontal mid L | -40 04 60 | 8.63 | 70 | <0.0001 |
|  | Frontal mid R, Frontal sup R, | 46 50 08 | 8.26 | 276 | <0.0001 |
|  | Cereb 9 R, Cereb 9 L | 02 -58 -54 | 8.22 | 232 | <0.0001 |
|  | Frontal mid R, Frontal inf tri R | 38 36 20 | 8.21 | 159 | <0.0001 |
|  | Precentral L | -40 -04 40 | 8.21 | 136 | <0.0001 |
|  | Cereb Crus 1 R, Cereb 6 R | 40 -74 -26 | 8.13 | 448 | <0.0001 |
|  | Putamen R, Pallidum R | 22 02 10 | 8.05 | 112 | <0.0001 |
|  | Temporal pole sup R, OFCpost R | 32 22 -28 | 7.96 | 87 | <0.0001 |
|  | Frontal sup medial L | -12 68 18 | 7.69 | 31 | <0.0001 |
|  | Frontal sup L, | -16 24 56 | 7.06 | 109 | <0.0001 |
|  | Temporal inf L | -64 -18 -32 | 7.04 | 25 | 0.001 |
|  | Tempoal inf R | 66 -10 -32 | 6.99 | 56 | <0.0001 |
|  | Frontal med Orb L, Frontal med Orb R, Rectus L | 0 66 -16 | 6.98 | 223 | <0.0001 |
|  | Postcentral R, Temporal sup R, Supramarginal R, Rolandic Oper R | 62 -16 18 | 6.91 | 245 | <0.0001 |
|  | Cereb 8 L | -26 -46 -60 | 6.69 | 23 | 0.001 |
|  | Frontal inf Orb R, Temporal pole sup R | 54 22 -12 | 6.51 | 43 | <0.0001 |
|  | Frontal sup R | 22 10 70 | 6.43 | 22 | 0.001 |
|  | SNc l, SNr L | -10 -16 -16 | 6.33 | 27 | <0.0001 |
|  | Amygdala R | 24 02 -14 | 6.25 | 28 | <0.0001 |
|  | Frontal sup R | 20 36 42 | 6.15 | 26 | <0.0001 |
|  | Putamen L, Thal VA L | -16 -02 12 | 6.00 | 49 | <0.0001 |
|  | SNc R, SNr R | 10 -16 -16 | 5.99 | 29 | <0.0001 |
|  | Frontal inf tri R, Frontal inf oper R, Frontal mid R | 52 22 30 | 5.98 | 50 | <0.0001 |
|  | Frontal sup L | -16 44 30 | 5.82 | 27 | <0.0001 |
|  | Lingual R | 30 -66 02 | 5.75 | 24 | 0.001 |
|  | Cereb 7 L, Cereb Crus 2 L, Cereb 8 | -20 -76 -48 | 5.69 | 52 | <0.0001 |
|  | Frontal sup medial L | -10 58 10 | 5.39 | 30 | <0.0001 |
| HC<PD | Precuneous L, Precuneous R, Paracentral Lobule L, SMA L, SMA R, Paracentral lobe R, Cingulate mid R, Cingulate mid L, Precentral R, Precentral L, Postcentral L, Postcentral R, Frontal mid R | 0 -48 56 | 18.13 | 13172 | <0.0001 |
|  | Frontal mid L | -24 36 22 | 9.42 | 114 | <0.0001 |
|  | Occipital mid L, | -20 -88 16 | 9.23 | 284 | <0.0001 |
|  | Lingual L | -04 -96 -20 | 8.95 | 47 | <0.0001 |
|  | OFCant R | 22 60 -20 | 8.58 | 96 | <0.0001 |
|  | Occipital Sup R, Calcarine R | 20 -80 18 | 8.15 | 185 | <0.0001 |
|  | OFCmed L | -18 58 -20 | 7.74 | 32 | <0.0001 |
|  | NAcc R, Olfactory R | 08 14 -08 | 7.69 | 193 | <0.0001 |
|  | Frontal Sup R | 24 44 18 | 7.63 | 67 | <0.0001 |
|  | Putamen R, Insula R | 36 -10 02 | 7.61 | 258 | <0.0001 |
|  | Fusiform L, Temporal inferior L | -38 -26 -30 | 7.57 | 338 | <0.0001 |
|  | Frontal Sup medial L | 0 48 46 | 7.38 | 42 | <0.0001 |
|  | Frontal Sup L | -18 60 30 | 7.36 | 28 | <0.0001 |
|  | Vermis 3 | -02 -38 -10 | 7.09 | 99 | <0.0001 |
|  | Putamen L | -32 -08 04 | 6.68 | 70 | <0.0001 |
|  | Parietal sup L, Parietal inf L | -38 -62 58 | 6.66 | 72 | <0.0001 |
|  | OFCant L | -26 34 -18 | 6.64 | 93 | <0.0001 |
|  | Temporal sup R | 60 -24 06 | 6.52 | 47 | <0.0001 |
|  | Cereb 4,5 L | -10 -50 -18 | 6.38 | 113 | <0.0001 |
|  | Occipital sup R | 28 -88 30 | 6.33 | 34 | <0.0001 |
|  | Angular R | 26 -60 40 | 6.27 | 34 | <0.0001 |
|  | OFCmed R | 22 22 -18 | 5.84 | 23 | 0.001 |
|  | Lingual L | -08 -64 -04 | 5.71 | 54 | <0.0001 |
|  | Fusiform R | 38 -06 -42 | 5.55 | 23 | 0.001 |

Supplementary Table 2: Hypometabolic and hypermetabolic clusters in PwPD relative to healthy controls based on metabolic time series comparison using a subject x Time Design in SPM12. Threshold: p<0.05 after Cluster-level FWE correction.

Abbreviations (AALv3):

| **Abbreviation** | **Full Name** |
| --- | --- |
| **Amygdala R** | Amygdala right |
| **Angular L** | Angular gyrus left |
| **Angular R** | Angular gyrus right |
| **Calcarine L** | Calcarine cortex left |
| **Calcarine R** | Calcarine cortex right |
| **Cereb 8 L** | Cerebellum Lobule VIII left |
| **Cereb 9 L** | Cerebellum Lobule IX left |
| **Cereb 9 R** | Cerebellum Lobule IX right |
| **Cereb Crus 1 R** | Cerebellum Crus I right |
| **Cereb Crus 2 L** | Cerebellum Crus 2 left |
| **Cereb 7 L** | Cerebellum Lobule VII left |
| **Cerebellum 8** | Cerebellum Lobule VIII |
| **Cereb Crus 1** | Cerebellum Crus I |
| **Cereb 4** | Cerebellum Lobule IV |
| **Cereb 6** | Cerebellum Lobule VI |
| **Cingulate mid L** | Middle cingulate cortex left |
| **Cingulate mid R** | Middle cingulate cortex right |
| **Cuneus L** | Cuneus left |
| **Frontal Sup L** | Superior frontal gyrus left |
| **Frontal Sup medial L** | Medial superior frontal gyrus left |
| **Frontal Sup R** | Superior frontal gyrus right |
| **Frontal inf Orb R** | Inferior frontal gyrus (orbital part) right |
| **Frontal inf oper L** | Inferior frontal gyrus (opercular part) left |
| **Frontal inf oper R** | Inferior frontal gyrus (opercular part) right |
| **Frontal inf tri L** | Inferior frontal gyrus (triangular part) left |
| **Frontal inf tri R** | Inferior frontal gyrus (triangular part) right |
| **Frontal med Orb L** | Medial orbitofrontal cortex left |
| **Frontal med Orb R** | Medial orbitofrontal cortex |
| **Frontal mid L** | Middle frontal gyrus left |
| **Frontal mid R** | Middle frontal gyrus right |
| **Frontal sup R** | Superior frontal gyrus right |
| **Frontal sup L** | Superior frontal gyrus left |
| **Frontal sup medial L** | Medial superior frontal gyrus left |
| **Fusiform L** | Fusiform gyrus left |
| **Fusiform R** | Fusiform gyrus right |
| **Insula L** | Insula left |
| **Insula R** | Insula right |
| **Lingual L** | Lingual gyrus left |
| **Lingual R** | Lingual gyrus right |
| **NAcc R** | Nucleus accumbens right |
| **OFC post L** | Orbitofrontal cortex posterior part left |
| **OFCant L** | Orbitofrontal cortex anterior part left |
| **OFCant R** | Orbitofrontal cortex anterior part right |
| **OFCmed L** | Orbitofrontal cortex medial part left |
| **OFCmed R** | Orbitofrontal cortex medial part right |
| **OFCpost R** | Orbitofrontal cortex posterior part right |
| **Occipital inf L** | Inferior occipital gyrus left |
| **Occipital inf R** | Inferior occipital gyrus right |
| **Occipital mid L** | Middle occipital gyrus left |
| **Occipital mid R** | Middle occipital gyrus right |
| **Occipital sup L** | Superior occipital gyrus left |
| **Occipital sup R** | Superior occipital gyrus right |
| **Olfactory R** | Olfactory cortex right |
| **Pallidum R** | Pallidum right |
| **Paracentral lobule R** | Paracentral lobule right |
| **Paracentral lobule L** | Paracentral lobule left |
| **Parietal inf L** | Inferior parietal lobule left |
| **Parietal sup L** | Superior parietal lobule left |
| **Postcentral L** | Postcentral gyrus left |
| **Postcentral R** | Postcentral gyrus right |
| **Precentral L** | Precentral gyrus left |
| **Precentral R** | Precentral gyrus right |
| **Precuneous r** | Precuneus right |
| **Precuneous L** | Precuneus left |
| **Putamen L** | Putamen left |
| **Putamen R** | Putamen right |
| **Rectus L** | Gyrus rectus left |
| **Rolandic Oper R** | Rolandic operculum right |
| **SMA L** | Supplementary motor area left |
| **SMA R** | Supplementary motor area right |
| **SNc L** | Substantia nigra pars compacta left |
| **SNc R** | Substantia nigra pars compacta right |
| **SNr L** | Substantia nigra pars reticulata left |
| **SNr R** | Substantia nigra pars reticulata right |
| **SupraMarginal L** | Supramarginal gyrus left |
| **SupraMarginal R** | Supramarginal gyrus right |
| **Temporal inf R** | Inferior temporal gyrus right |
| **Temporal inf L** | Inferior temporal gyrus left |
| **Temporal inferior L** | Inferior temporal gyrus left |
| **Temporal pole sup L** | Superior temporal pole left |
| **Temporal pole sup R** | Superior temporal pole right |
| **Temporal mid L** | Middle temporal gyrus left |
| **Temporal mid R** | Middle temporal gyrus right |
| **Temporal sup R** | Superior temporal gyrus right |
| **Vermis 3** | Cerebellar vermis lobule III |

| Group | Seed | MNI-coordinates | t-value | cluster size  (number of voxels) | P_FWE_ |
| --- | --- | --- | --- | --- | --- |
| fMRI | | | | | |
| HC | SN L | -09 -15 -15 |  | 41 | 0.000046 |
| PD | SN L | -09 -15 -15 |  | 21 | 0.000001 |
| HC | SN R | 09 -15 -15 |  | 43 | 0.000002 |
| PD | SN R | 09 -15 -15 |  | 49 | 0.000104 |
| PD | SN R | 63 -33 00 |  | 32 | 0.028830 |
| fPET | | | | | |
| HC | SN L | -08 -16 -16 | 116.66 | 256 | 0.0000 |
| PD | SN L | -10 -16 -16 | 80.95 | 304 | 0.0000 |
| HC | SN R | 10 -16 -16 | 103.15 | 256 | 0.0000 |
| PD | SN R | 10 -16 -16 | 103.58 | 300 | 0.0000 |

**Supplementary Table 3: Group-level seed-to-voxel results using the data-driven obtained hypometabolic cluster as seed.** Threshold: p_FWE_<0.05 at peak level. Abbreviations: SN L = Substantia nigra left, SN R = Substantia nigra right


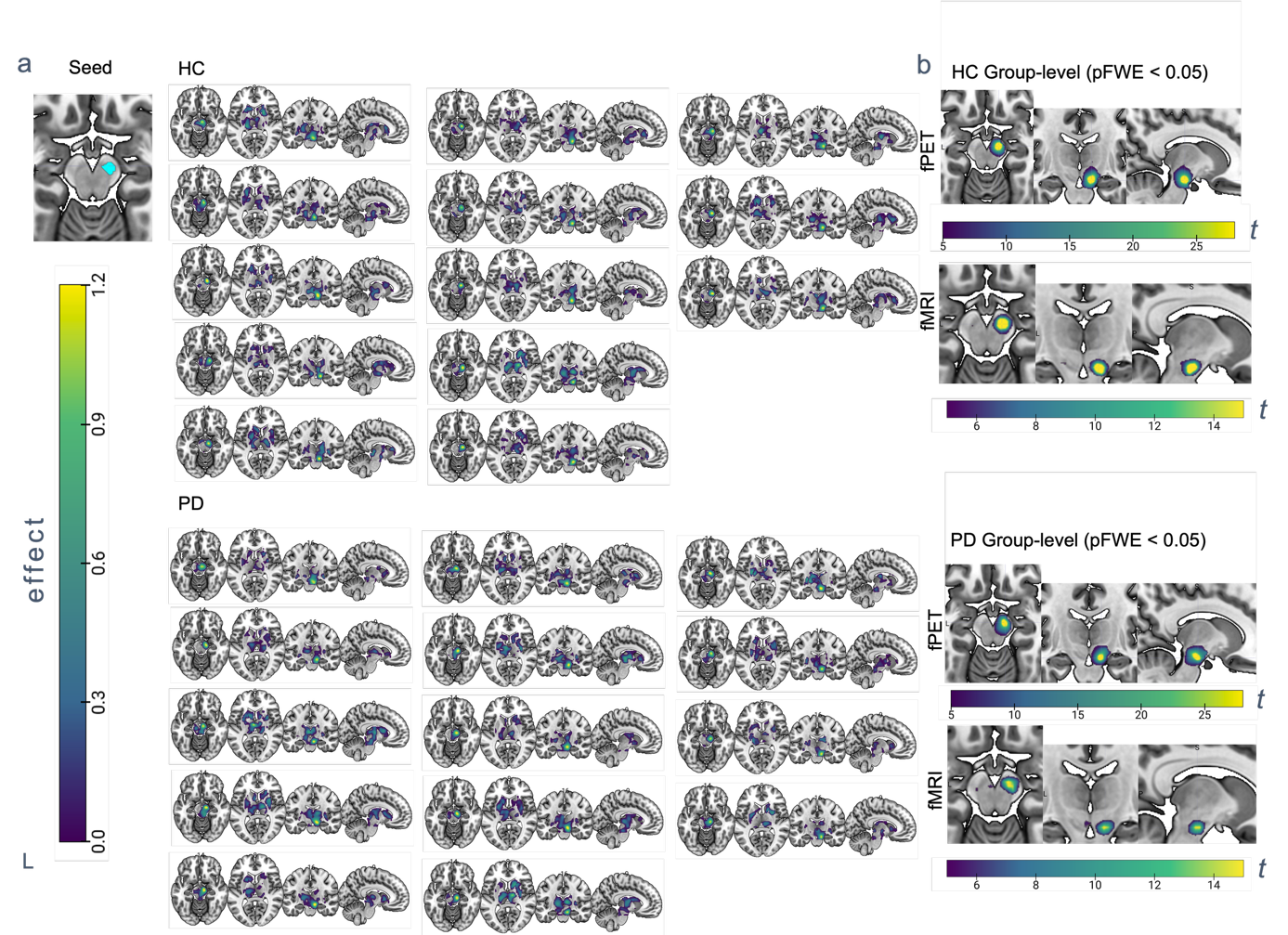


**Fig. S1:** **Seed-based nigral networks in PwPD and healthy controls on subject- and group-level.** SPM result of first-level single-subject regression analysis using the right substantia nigra as seed visualised on MNI standard template. Sagittal, coronal and axial view of regions of (a) subject-level contrast images or (b) group-level *t*-maps obtained by voxel-wise analysis of [^18^F]-FDG PET scans from 13 healthy controls and 14 PD patients (P < 0.05 after FWE cluster level correction). Color bar represents subject-level effect in contrast images (a) or *t*-values of a voxel wise one-sample *t*-test of contrast images (b). All images are shown in neurological displa


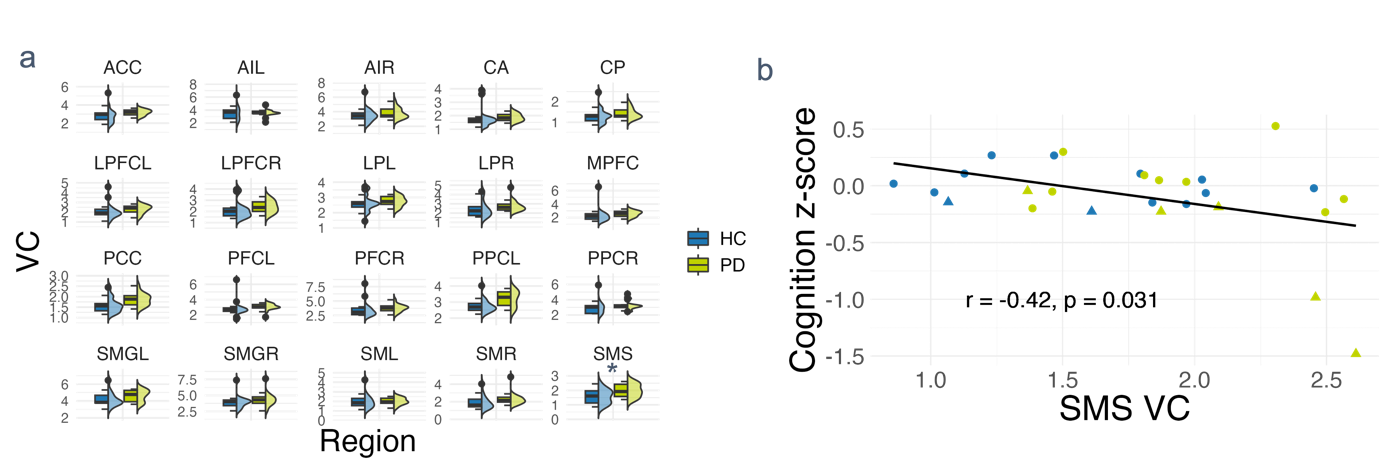


**Fig. S2: Metabolic time series variation in PwPD relative to healthy controls.** Subject-level variation coefficients from extracted metabolic time series in canconical resting-state network nodes. Marked dots represent outliers with values > 1.5 times the inter-quartile distance. b showes a scatterplot relating regional variation coefficients to cognitive performance measured by cognition z-scores. The subjects marked in rectanglular shape represent the subjects classified as MCI. Abbreviations: VC = variation coefficient

| Network name | Seed | x | | y | z |
| --- | --- | --- | --- | --- | --- |
| Default mode network (DMN) | Medial prefrontal cortex  Latral parietal cortex (L)  Lateral parietal cortex (R)  Posterior cingulate cortex | 1  -39  47  1 | 55  -77  -67  -61 | | -3  33  29  38 |
|  |  |  |  | |  |
| Frontoparietal network (FPN) | Lateral prefrontal cortex (L)  Posterior parietal coretx (L)  Lateral prefrontal cortex (R)  Posterior parietal cortex (R) | -43  -46  41  52 | 33  -58  38  -52 | | 28  49  30  45 |
| Salience network (SN) | Anterior cingulate cortex  Anterior insula (L)  Anterior insula (R)  Rostral prefrontal cortex (L)  Rostral prefrontal cortex (R)  Supramarginal gyrus (L)  Supramarginal gyrus (R) | 0  -44  47  -35  32  -60  62 | 22  13  14  45  46  -39  -35 | | 35  1  0  27  27  31  32 |

**Supplementary Table 4. CONN toolbox network region of interest (ROI) definitions.**

| **Test** | **HC (n=11)** | **PD-MCI(n=5)** | **PD-NC(n=9)** | **statistic** | **p-value** |
| --- | --- | --- | --- | --- | --- |
| z_RWT_SKW_sport_fruits | 0.05 ± 0.99 | -1.40 ± 1.60 | 0.17 ± 1.05 | 4.02 | 0.134 |
| z_PANDA_WFL_t | -0.01 ± 1.03 | -0.95 ± 1.38 | 0.16 ± 1.22 | 3.05 | 0.217 |
| z_WMSR_ds_f_raw | -0.09 ± 0.98 | -0.89 ± 0.99 | 0.73 ± 1.05 | 6.04 | **0.049^*1^** |
| z_WMSR_ds_b_raw | 0.22 ± 0.91 | -1.17 ± 0.92 | -0.02 ± 1.09 | 5.72 | 0.057 |
| z_WMSR_ss_b_raw | 0.16^c^ ± 0.86 | -1.89 ± 2.54 | 0.32 ± 1.34 | 4.08 | 0.130 |
| z_WMSR_ss_f_raw | 0.16 ± 0.93 | -0.96 ± 2.47 | -0.23 ± 1.83 | 0.70 | 0.704 |
| z_PANDA_relayed recall | 0.09 ± 1.01 | -1.30 ± 1.58 | 0.11 ± 0.95 | 5.59 | 0.061 |
| z_MoCa_language | -0.12 ± 1.05 | -1.02 ± 0.93 | -0.52 ±1.10 | 2.59 | 0.274 |
| z_RWT_SK_animals | 0.17 ± 0.93 | -0.77 ± 1.43 | -0.16 ± 1.08 | 2.18 | 0.337 |
| z_PANDA_spatial | 0.32 ± 0.67 | 0.06 ± 0.89 | 0.38 ± 0.96 | 1.40 | 0.496 |
| Cog_z | 0.08 ± 0.36^c^ | -1.44 ± 1.52^a^ | 0.11 ± 0.60 | 7.10 | **0.029^*2^** |

**Supplementary Table 5: Neuropsychological test results of patients undergoing fPET scanning and neuropsychological testing.** Domain-specific tests were compared between all groups using Kruskal-Wallis test and pair-wise Wilcoxon tests were used to evaluate individual between-group differences.^*1^ Kruskal-Wallis effect size: eta^2^ = 0.17 (large), ^*2^ Kruskal-Wallis effect size: eta^2^ = 0.21 (large).

| a fMRI | | | | | | | |
| --- | --- | --- | --- | --- | --- | --- | --- |
| Group | Seed | | Regions | MNI-coordinates (x,y,z) | Size  (number of voxels) | Z-Values | p-FWE Cluster-level |
| HC | SMS | | Postcentral L, Parietal Sup L, Precuneus L, Precuneus R, Postcentral R, Precentral R, Frontal Sup R, SMA L, SMA R, Frontal Sup L, Precentral L, SupraMarginal L, Rolandic Oper L, Heschl L, Cingulate Mid L, Cingulate Mid R,Heschl R, Teporal Sup R, SupraMarginal R, Rolandic Oper R, Temporal Sup R | -27 -42 66 | 9058 |  | 0.000000 |
|  |  | | Cereb2 L,Cereb1 L, Cereb8 L, Cereb7 L, Cereb6 L, Cereb9 L | -24 -72 -27 | 803 |  | 0.000000 |
|  |  | | Cereb1 R, Cereb2 R, Cereb6 R, Cereb8 R, Cereb7 R | 27 -54 -33 | 484 |  | 0.004000 |
|  |  | | Lingual L, Calcarine L | -18 -57 03 | 350 |  | 0.012000 |
|  |  | | Parietal Inf L, | -45 -48 36 | 317 |  | 0.016000 |
|  |  | | Lingual R, Calcarine R | 15 -54 03 | 264 |  | 0.034000 |
|  |  | | Angular R, Parietal Inf R, SupraMarginal R | 42 -66 51 | 225 |  | 0.047000 |
| PD-NC | SMS | | Precuneus L, Postcentral R, Parietal Sup R, Precuneus L, Postcentral L, Precentral L, SMA L, SMA R, Frontal Sup R, Frontal Sup L, Paracentral Lobule L, Paracentral Lobule R, Heschl L, Insula L, Heschl R, Insula R, SupraMarginal R, Parietal Inf L, SupraMarginal L, Putamen L, Rolandic Oper L, Putamen R | -06 -42 69 | 5582 |  | 0.000000 |
|  |  | | Cereb2 R, Cereb2 L, Cereb1 R, Cereb1 L, Cereb7 R, Cereb8 R | 30 -81 -39 | 813 |  | 0.007000 |
| PD-MCI | SMS | | Paracentral Lobule R, SMA L, SMA R, Paracentral Lobule L, Precentral L, Precentral R, Frontal Sup R, Frontal Sup L, Postcentral L, Postcentral R, Precuneus L, Precuneus R, Parietal Sup L, Parietal Sup R | 09 -36 66 | 2495 |  | 0.000000 |
|  |  | | Calcarine R, Lingual R, Calcarine L | 12 -57 12 | 413 |  | 0.023000 |
| HC | ACC | | Mid Cingulate R, ACC sup L, Frontal Sup Medial L, SMA L, Cingulate Mid L, ACCpre L, Frontal Mid L, SMA R, Cingulate Mid R, Frontal Sup Medial R | 03 24 30 | 3550 |  | 0.000000 |
|  |  | | Frontal Inf Oper R, Temporal Pole Sup R, Insula R, Putamen R, Caudate R, | 42 15 12 | 1025 |  | 0.006000 |
|  |  | | Insula L, Temporal Pole Sup L, Putamen L, Frontal Inf Tri L, Thal VA L, Rolandic Oper L | -33 09 -12 | 983 |  | 0.006000 |
|  |  | | Thal IL R | 21 -39 30 | 687 |  | 0.013000 |
|  |  | | Cereb2,  Cereb1 R,  Cereb7 R, Cereb2 L,  Cereb8 R | 42 -75 -45 | 437 |  | 0.024000 |
| PD-NC | ACC | | SupraMarginal L, Postcentral L, Temporal Sup L, Rolandic Oper L, Putamen R, Frontal Inf Oper R, Insula R, Heschl R, Putamen L, Insula L, Temporal Pole Mid L | -60 -33 27 | 2177 |  | 0.000000 |
|  |  | | Cingulate Mid R, ACCsup R, SMA R, SMA L, ACCsup R,ACCpre L, Postcentral R, Precentral R | 03 -15 45 | 1715 |  | 0.000000 |
| PD-MCI | ACC | | Cingulate Mid R, Cingulate Mid L, ACC sup L, Frontal Sup Medial L, SMA L, SMA R, ACCpre L, Frontal sup Medial R, Frontal sup L, Frontal Mid L, ACCpre R | -03 18 51 | 1762 |  | 0.000000 |
| HC | PCC | | Precuneus R, Precuneus L, Parietal Sup L,Parietal Inf L, Occipital Mid L, Angular L, Temporal Mid L, Parietal Sup R, Occipital Sup R, Angular R, Occipital Mid R, Cuneus R, Calcarine R, Calcarine L, Lingual L, Lingual R, Cingulate Mid L, Cingulate Post L, ThalPuM L, Cingulate Post R, Cingulate Mid R | 06 -69 51 | 6092 |  | 0.000000 |
|  |  |  | Frontal Inf Orb R, Frontal Mid R, Frontal Inf Tri R, OFClat R, | 48 42 -12 | 1192 |  | 0.001000 |
|  |  |  | Frontal Sup R, Frontal Sup Medial R, ACCPre L, ACCPre R, Frontal Med Orb R, ACC Sup R, | 18 60 06 | 665 |  | 0.004000 |
|  |  |  | SMA R, SMA L | 03 -12 60 | 595 |  | 0.005000 |
|  |  |  | Precentral L | -39 -06 36 | 518 |  | 0.009000 |
|  |  |  | Cereb9 R, Cereb9 L, Cereb8 R,  Cereb7 R, Cereb2 R | 09 -57 -48 | 245 |  | 0.049000 |
| PD-NC | PCC | | Precuneus L, Calcarine L, Cingulate Post L, Cingulate Mid L, Cuneus L, Cuneus R, Precuneus R, Calcarine R, Lingual R, Cingulate Mid R, Cingulate Post R, | 00 -54 57 | 3112 |  | 0.000000 |
|  |  |  | Angular L, Parietal Sup L, Occipital Mid L, Temporal Mid L | -51 -66 42 | 546 |  | 0.010000 |
|  |  |  | Temporal Pole Mid R, Temporal Inf R | 45 06 -33 | 221 |  | 0.047000 |
| PD-MCI | PCC | | Calcarine R, Lingual R, Cuneus R | 15 -93 21 | 1825 |  | 0.000000 |
|  |  |  | Heschl R, Insula R, Rolandic Oper R, Temporal Sup R | 42 -24 12 | 505 |  | 0.0000 |
|  |  |  | Insula L, Frontal Inf Tri L, Frontal Inf Oper L | -39 03 12 | 241 |  | 0.046000 |
| HC | LPFC-R | | Frontal Mid R, Frontal Sup R, Frontal Inf Tri R, Frontal Inf Oper R, Precentral R, OFClat R, OFCant R, OFCmed R | 30 48 03 | 3113 |  | 0.000000 |
|  |  |  | Frontal Mid L, Frontal Sup L, Frontal Inf Tri L, OFCantL, OFClat L, Frontal Inf Oper L | -42 51 -06 | 1279 |  | 0.000000 |
|  |  |  | Cerebellum 4_5, Hippocampus L, Lingual L, Calcarine L | -21 -33 -24 | 1022 |  | 0.000000 |
|  |  |  | Postcentral L, Precentral L, Parietal Sup L, Parietal Inf L, Precuneus L, Frontal Sup L, SMA L | -30 -42 63 | 814 |  | 0.000000 |
|  |  |  | Parietal Inf R, SupraMarginal R, Angular R | 42 -51 48 | 810 |  | 0.000000 |
|  |  |  | Cereb2 L, Cereb 1 L, Cereb7 L, Cereb 8 L | -42 -60 -45 | 591 |  | 0.006000 |
|  |  |  | Parietal Inf L, SupraMarginal L, Parietal Sup L | -57 -54 42 | 522 |  | 0.011000 |
| PD-NC | LPFC-R | | Frontal Sup Medial R, Frontal Sup Medial L, Cingulate Mid R, Frontal Sup R | 06 30 45 | 1343 |  | 0.000000 |
|  |  |  | Parietal Inf R, Angular R, Parietal Sup R | 45 -54 45 | 510 |  | 0.001000 |
|  |  |  | Cereb2 L, Cereb 1 L, Cereb 7 L | -21 -78 -51 | 365 |  | 0.014000 |
| PD-MCI | LPFC-R | | Frontal Sup R, Frontal Mid R, Frontal Inf Tri R | 24 15 66 | 621 |  | 0.004000 |
| b fPET | | | | | | | |
| HC | | SMS | Frontal Sup Medial L, SMA L, Fromtal Supp L | -04 34 54 | 868 | 5.07 | 0.000 |
|  |  |  | Paracentral Lobule R, Paracentral Lobule L, Precentral L, Postcentral L, SMA R, Cingulate Mid R | 0 -26 66 | 2138 | 5.04 | 0.000 |
|  |  |  | SupraMarginal L, Parietal inferior L, Temporal Sup L | -56 -44 32 | 626 | 4.93 | 0.000 |
|  |  |  | Cingulate Mid R, SMA R | 10 16 44 | 254 | 4.91 | 0.000 |
|  |  |  | Precentral L, Postcentral L | -50 -4 32 | 256 | 4.86 | 0.000 |
|  |  |  | Frontal Sup R | 20 20 58 | 249 | 4.85 | 0.000 |
|  |  |  | Frontal Mid R | 36 52 -12 | 137 | 4.71 | 0.005 |
|  |  |  | Precuneus R | 08 -54 26 | 478 | 4.65 | 0.000 |
|  |  |  | Putamen L | -22 06 04 | 547 | 4.54 | 0.000 |
|  |  |  | Rolandic Operculum R | 62 02 12 | 156 | 4.48 | 0.002 |
|  |  |  | Precentral R, Postcentral R | 20 -24 76 | 237 | 4.36 | 0.000 |
|  |  |  | Temporal Mid L | -58 -58 08 | 87 | 4.29 | 0.048 |
|  |  |  | Frontal Mid R, Frontal Sup R | 30 42 16 | 101 | 4.13 | 0.025 |
|  |  |  | Frontal Sup L, Frontal Sup Medial L | -12 56 22 | 213 | 4.04 | 0.000 |
|  |  |  | Frontal Sup R, Frontal Sup Medial R | 16 58 24 | 95 | 4.02 | 0.033 |
| PD-NC | | SMS | Frontal Mid R, Precentral R | 40 08 44 | 137 | 5.24 | 0.000 |
|  |  |  | Postcentral R, Parietal sup R, Paracentral Lobule R, SMA R, Paracentral Lobule L, SMA L, Cingulate Mid R | 14 -38 72 | 2023 | 5.21 | 0.000 |
|  |  |  | Precuneus R, Cuneus R | 16 -64 28 | 250 | 5.00 | 0.000 |
|  |  |  | Frontal Inf Tri L, Frontal Mid L | -46 32 10 | 473 | 4.89 | 0.000 |
|  |  |  | Occipital Mid R, Occipital sup R | 32 -68 34 | 236 | 4.83 | 0.000 |
|  |  |  | Frontal Inf Tri R, Frontal Inf Oper R | 54 20 24 | 112 | 4.82 | 0.002 |
|  |  |  | Precuneus L | -06 -62 48 | 119 | 4.67 | 0.001 |
|  |  |  | Cingulate post R, Cingulate Mid R | 10 -40 28 | 243 | 4.63 | 0.000 |
|  |  |  | Frontal sup R, Frontal Mid R | 22 44 38 | 450 | 4.62 | 0.000 |
|  |  |  | Frontal Inf Tri R, Frontal Inf oper R | 50 22 02 | 101 | 4.53 | 0.004 |
|  |  |  | Frontal sup R | 26 54 20 | 73 | 4.49 | 0.025 |
|  |  |  | Temporal Mid L | -58 -60 18 | 140 | 4.40 | 0.000 |
|  |  |  | Frontal Mid R, Frontal sup R | 40 52 10 | 152 | 4.35 | 0.000 |
|  |  |  | Temporal sup L, Postcentral L, SupraMarginal L | -60 -32 22 | 114 | 4.33 | 0.002 |
|  |  |  | Angular L, Temporal Mid L | -48 -64 34 | 156 | 4.24 | 0.000 |
|  |  |  | Paracentral Lobule L, Postcentral L | -18 -24 72 | 244 | 4.18 | 0.000 |
|  |  |  | SupraMarginal R | 62 -38 28 | 151 | 4.17 | 0.000 |
|  |  |  | SupraMarginal R, Postcentral R | 56 -26 40 | 235 | 4.17 | 0.000 |
|  |  |  | Putamen L, Pallidum L | -24 06 -04 | 117 | 4.14 | 0.002 |
|  |  |  | Fronta Mid L, Frontal Inf Oper L, Precentral L | -32 20 44 | 113 | 4.07 | 0.002 |
|  |  |  | Occipital sup L, Cuneus L | -22 -74 34 | 106 | 3.97 | 0.003 |
|  |  |  | OFCpost R, OFCant R | 36 34 -14 | 121 | 3.94 | 0.001 |
|  |  |  | Postcentral L, Parietal sup L | -28 -44 68 | 79 | 3.77 | 0.017 |
|  |  |  | Precentral R | 32 -14 54 | 84 | 3.69 | 0.012 |
| PD-MCI | | SMS | Paracentral Lobule L, SMA L | -08 -22 62 | 53 | 4.17 | 0.003 |
|  |  |  | Putamen R | 26 06 -04 | 35 | 4.11 | 0.037 |
|  |  |  | Parietal Inf R, SupraMarginal R | 44 -44 44 | 53 | 4.10 | 0.003 |
|  |  |  | Cuneus R, Precuneus R | 10 -74 36 | 60 | 4.10 | 0.001 |
|  |  |  | Precentral R | 20 -16 76 | 46 | 3.88 | 0.008 |
|  |  |  | Temporal sup R | 60 -08 02 | 39 | 3.87 | 0.021 |
|  |  |  | Fontal Mid R, | 28 36 36 | 41 | 3.78 | 0.015 |
|  |  |  | Parietal sup L | -24 -60 56 | 47 | 3.78 | 0.007 |
| HC | | ACC | Cingulate Mid R, SMA L, ACC sup R, ACC sup L, Frontal sup Medial L, Cingulate Mid L, ACC sup R | 06 14 40 | 1308 | 5.96 | 0.000 |
|  |  |  | Thal VL R, Thal VPL R | 12 -16 06 | 89 | 4.79 | 0.039 |
|  |  |  | Calcarine R | 20 -70 08 | 105 | 4.41 | 0.018 |
|  |  |  | Frontal Inf Tri L, Frontal Inf Oper L | -52 14 24 | 104 | 4.09 | 0.019 |
|  |  |  | Frontal Sup L, Frontal Mid L | -20 30 44 | 98 | 4.01 | 0.025 |
|  |  |  | Occipital Mid L, Angular L, Temporal Mid L | -44 -72 24 | 94 | 3.91 | 0.030 |
| PD-NC | | ACC | Frontal sup Medial L, Cingulate Mid R, ACC sup R, SMA L, SMA R, ACC sup L, Cingulate Mid L | 04 12 42 | 1175 | 5.85 | 0.000 |
|  |  |  | Putamen R | 22 14 -02 | 77 | 4.72 | 0.016 |
|  |  |  | Precunus R, Precuneus L | 10 -52 30 | 105 | 4.50 | 0.002 |
|  |  |  | OFCpost L, Frontal Inf Orb L | -18 32 -12 | 71 | 4.24 | 0.024 |
|  |  |  | Frontal Mid L | -36 06 36 | 66 | 3.97 | 0.034 |
| PD-MCI | | ACC | Cingulate Mid R, SMA R, Cingulate Mid L, SMA L | 06 22 36 | 363 | 4.32 | 0.000 |
|  |  |  | Fusiform R | 36 -38 -16 | 28 | 3.92 | 0.044 |
| HC | | PCC | Precuneus L, Calcarine L, Precuneus R, Cuneus L | -12 -68 56 | 2852 | 5.25 | 0.000 |
|  |  |  | Thalamus PuA L, Thalamus VPL L, Thalamus PuM L, Thalamus IL L | -12 -24 06 | 103 | 4.83 | 0.024 |
|  |  |  | Temporal Mid L, Angular L | -56 -56 10 | 486 | 4.64 | 0.000 |
|  |  |  | Postcentral L, Precentral L | -22 -32 64 | 273 | 4.63 | 0.000 |
|  |  |  | SupraMarginal R, Angular R,Parietal Inf R | 64 -44 32 | 224 | 4.46 | 0.000 |
|  |  |  | Angular R, Occipital Mid R | 42 -74 44 | 99 | 4.17 | 0.029 |
|  |  |  | Precentral R, Frontal Mid R, Frontal Sup R | 38 -08 56 | 93 | 4.05 | 0.039 |
| PD-NC | | PCC | Precuneus L, Precuneus R, Cingulate Mid R, Cingulate Mid R, Cuneus L, Cuneus R | -02 -46 40 | 2831 | 5.78 | 0.000 |
|  |  |  | Frontal Inf Tri R, Frontal Inf Oper R, Insula R | 50 22 02 | 305 | 5.14 | 0.000 |
|  |  |  | SupraMarginal R, Postcentral R | 58 -24 36 | 281 | 4.59 | 0.000 |
|  |  |  | Frontal Inf Tri L, Frontal Mid L | -36 34 26 | 120 | 4.57 | 0.001 |
|  |  |  | ACCpre R, Frontal sup Medial R | 10 50 12 | 86 | 4.52 | 0.011 |
|  |  |  | Thal VL R, Thal VPL R | 14 -16 -04 | 72 | 4.51 | 0.027 |
|  |  |  | Parietal Inf R, Angular R | 46 -56 48 | 116 | 4.42 | 0.002 |
|  |  |  | Occipital Inf L, Fusiform L | -46 -72 -12 | 71 | 4.36 | 0.029 |
|  |  |  | Calcarine R, Lingual R | 28 -64 16 | 93 | 4.33 | 0.007 |
|  |  |  | Frontal sup Medial R, Frontal sup R, Frontal Mid R | 10 30 40 | 137 | 4.22 | 0.000 |
|  |  |  | Temporal sup L | -44 -12 -08 | 88 | 4.14 | 0.009 |
|  |  |  | OFCant R, OFC lat R | 28 40 -14 | 70 | 4.08 | 0.031 |
|  |  |  | Precentral R | 36 -12 54 | 77 | 4.06 | 0.019 |
|  |  |  | Temporal Mid L | -56 -60 26 | 160 | 4.93 | 0.000 |
|  |  |  | Frontal sup R | 30 56 10 | 109 | 3.90 | 0.002 |
|  |  |  | SMA R, Frontal sup R, SMA L | 14 08 62 | 119 | 3.88 | 0.001 |
|  |  |  | Temporal Mid R, Temporal sup R | 64 -46 06 | 68 | 3.69 | 0.036 |
| PD-MCI | | PCC | SMA R, Frontal sup R | 14 06 52 | 85 | 4.42 | 0.000 |
|  |  |  | Precuneus L, Precuneus R | 0 -60 42 | 399 | 4.25 | 0.000 |
|  |  |  | Calcarine R | 10 -66 14 | 83 | 4.19 | 0.000 |
|  |  |  | Precentral R | 46 -08 50 | 38 | 4.07 | 0.013 |
|  |  |  | Postcentral R | 24 -44 68 | 31 | 3.93 | 0.041 |
|  |  |  | SupraMarginal R, Temporal sup R | 54 -48 24 | 55 | 3.64 | 0.001 |
| HC | | LPFC-R | Frontal Mid R, Frontal Inf Orb R, Frontal Inf Tri R, Frontal sup R | 42 56 12 | 824 | 5.13 | 0.000 |
|  |  |  | Frontal Mid R, Frontal Inf Tri R | 42 16 52 | 942 | 5.04 | 0.000 |
|  |  |  | Putamen L, Pallidum L, Insula L | -24 -02 02 | 411 | 5.03 | 0.000 |
|  |  |  | Precuneus R, Cuneus L, Precuneus L, Cuneus R | 06 -72 36 | 233 | 4.70 | 0.000 |
|  |  |  | Frontal sup L | -22 08 58 | 145 | 4.53 | 0.004 |
|  |  |  | SMA R, Frontal Sup R, Precentral R, Paracentral Lobule R | 16 -14 66 | 102 | 4.48 | 0.024 |
|  |  |  | Temporal Mid L | -56 -44 -08 | 163 | 4.43 | 0.002 |
|  |  |  | Postcentral L, SupraMarginal L, Temporal sup L | -58 -20 24 | 207 | 4.08 | 0.000 |
|  |  |  | Frontal Mid L | -36 38 36 | 372 | 4.08 | 0.000 |
|  |  |  | Temporal Mid R, Temporal sup R | 56 -38 -02 | 100 | 3.96 | 0.027 |
|  |  |  | Paracentral Lobule L, Precentral L | -14 -26 68 | 172 | 3.95 | 0.001 |
|  |  |  | Frontal Mid L, Frontal sup L | -22 50 28 | 114 | 3.93 | 0.014 |
|  |  |  | Frontal Inf Oper L, Frontal Inf Tri L | -58 06 12 | 178 | 3.88 | 0.001 |
| PD-NC | | LPFC-R | Frontal Mid R, Frontal sup R, Frontal Inf Tri R | 46 26 44 | 653 | 5.62 | 0.000 |
|  |  |  | Frontal Mid L, OFCant L | -32 50 -12 | 84 | 5.25 | 0.012 |
|  |  |  | Caudate L | -12 08 14 | 77 | 4.90 | 0.019 |
|  |  |  | Frontal sup R, Frontal Mid R | 32 56 06 | 489 | 4.76 | 0.000 |
|  |  |  | Frontal Inf Oper R, Insula R | 48 16 06 | 70 | 4.47 | 0.031 |
|  |  |  | Frontal Mid L, Frontal sup L | -32 36 22 | 170 | 4.34 | 0.000 |
|  |  |  | SupraMarginal R | 60 -34 30 | 134 | 4.25 | 0.001 |
|  |  |  | Angular R, Parietal sup R | 30 -60 50 | 82 | 4.00 | 0.014 |
|  |  |  | Temporal sup R | 66 -38 20 | 75 | 3.97 | 0.022 |
| PD-MCI | | LPFC-R | Frontal Mid R | 40 34 34 | 222 | 4.60 | 0.000 |
|  |  |  | Frontal Mid R, Frontal sup R | 42 54 06 | 64 | 4.27 | 0.000 |

Supplementary Table 6: Cluster wise statistical results per group revealed by seed-to-voxel connectivity analysis in a) CONN, b) SPM12. Threshold: p<0.05 after Cluster-level FWE correction.

Abbreviation (AALv3 Atlas):

| **Abbreviation** | Full name |
| --- | --- |
| **Frontal Sup R** | Frontal Sup, right |
| **Frontal Mid R** | Frontal Mid, right |
| **Cingulate Mid R** | Middle cingulate cortex, right |
| **SMA L** | Supplementary motor area, left |
| **SMA R** | Supplementary motor area, right |
| **Precuneus L** | Precuneus, left |
| **Postcentral L** | Postcentral gyrus, left |
| **Precentral R** | Precentral gyrus, right |
| **Precuneus R** | Precuneus, right |
| **Frontal Sup L** | Frontal Sup, left |
| **SupraMarginal R** | Supramarginal gyrus, right |
| **Frontal Mid L** | Frontal Mid, left |
| **Precentral L** | Precentral gyrus, left |
| **Postcentral R** | Postcentral gyrus, right |
| **Temporal Mid L** | Middle temporal gyrus, left |
| **Angular R** | Angular gyrus, right |
| **Calcarine R** | Calcarine cortex, right |
| **Cingulate Mid L** | Middle cingulate cortex, left |
| **Frontal Inf Tri R** | Frontall Inf Tri, right |
| **Parietal Sup L** | Superior parietal lobule, left |
| **Temporal Sup R** | Superior temporal gyrus, right |
| **Cuneus R** | Cuneus, right |
| **Frontal Inf Tri L** | Frontall Inf Tri, left |
| **Frontal Sup Medial L** | Frontall Sup Medial, left |
| **Frontal Sup Medial R** | Frontall Sup Medial, right |
| **Paracentral Lobule L** | Paracentral lobule, left |
| **SupraMarginal L** | Supramarginal gyrus, left |
| **Calcarine L** | Calcarine cortex, left |
| **Frontal Inf Oper R** | Frontall Inf Oper, right |
| **Insula R** | Insula, right |
| **Lingual R** | Lingual gyrus, right |
| **Parietal Inf L** | Inferior parietal lobule, left |
| **Parietal Inf R** | Inferior parietal lobule, right |
| **Parietal Sup R** | Superior parietal lobule, right |
| **Putamen L** | Putamen, left |
| **Angular L** | Angular gyrus, left |
| **Cereb2 L** | Cerebellum lobule II, left |
| **Cuneus L** | Cuneus, left |
| **Frontal Inf Oper L** | Frontal Inf Oper, left |
| **Insula L** | Insula, left |
| **Paracentral Lobule R** | Paracentral lobule, right |
| **Putamen R** | Putamen, right |
| **Temporal Sup L** | Superior temporal gyrus, left |
| **ACC Sup L** | Anterior cingulate cortex, left |
| **ACC Sup R** | Anterior cingulate cortex, right |
| **ACCpre L** | Anterior cingulate cortex, pregenual, left |
| **Cereb1 L** | Cerebellum lobule I, left |
| **Cereb7 R** | Cerebellum lobule VII, right |
| **Cereb8 R** | Cerebellum lobule VIII, right |
| **Heschl R** | Heschl's gyrus, right |
| **Rolandic Oper L** | Rolandic operculum, left |
| **ACCpre R** | Anterior cingulate cortex, pregenual, right |
| **Cereb1 R** | Cerebellum lobule I, right |
| **Cereb2 R** | Cerebellum lobule II, right |
| **Cereb7 L** | Cerebellum lobule VII, left |
| **Cingulate Post R** | Posterior cingulate cortex, right |
| **Lingual L** | Lingual gyrus, left |
| **OFCant R** | Orbitofrontal cortex, anterior, right |
| **OFClat R** | Orbitofrontal cortex, lateral, right |
| **Occipital Mid L** | Middle occipital gyrus, left |
| **Occipital Mid R** | Middle occipital gyrus, right |
| **ACCSup R** | Anterior cingulate cortex, right |
| **Cereb8 L** | Cerebellum lobule VIII, left |
| **Cereb9 L** | Cerebellum lobule IX, left |
| **Cingulate Post L** | Posterior cingulate cortex, left |
| **Frontal Inf Orb R** | Frontal Inf Orb, right |
| **Heschl L** | Heschl's gyrus, left |
| **OFCant L** | Orbitofrontal cortex, anterior, left |
| **Occipital Sup R** | Superior occipital gyrus, right |
| **Pallidum L** | Pallidum, left |
| **Rolandic Oper R** | Rolandic operculum, right |
| **Temporal Mid R** | Middle temporal gyrus, right |
| **Thal VL R** | Thalamus, ventral lateral nucleus, right |
| **Thal VPL R** | Thalamus, ventral posterolateral nucleus, right |
| **Caudate L** | Caudate nucleus, left |
| **Caudate R** | Caudate nucleus, right |
| **Cereb6 L** | Cerebellum lobule VI, left |
| **Cereb6 R** | Cerebellum lobule VI, right |
| **Cereb9 R** | Cerebellum lobule IX, right |
| **Cerebellum 4_5** | Cerebellum lobules IV–V |
| **Frontal Mid L** | Middle frontal gyrus, left |
| **Frontal Inf Orb L** | Frontall Inf Orb, left |
| **Frontal Inf oper R** | Frontal Inf oper, right |
| **Frontal Med Orb R** | Frontal Med Orb, right |
| **Frontal Sup L** | Frontall Supp, left |
| **Fusiform L** | Fusiform gyrus, left |
| **Fusiform R** | Fusiform gyrus, right |
| **Hippocampus L** | Hippocampus, left |
| **Mid Cingulate R** | Mid Cingulate, right |
| **OFClat L** | Orbitofrontal cortex, lateral, left |
| **OFCmed R** | Orbitofrontal cortex, medial, right |
| **OFCpost L** | Orbitofrontal cortex, posterior, left |
| **OFCpost R** | Orbitofrontal cortex, posterior, right |
| **Occipital Inf L** | Inferior occipital gyrus, left |
| **Occipital Sup L** | Superior occipital gyrus, left |
| **Rolandic Operculum R** | Rolandic operculum, right |
| **Temporal Inf R** | Inferior temporal gyrus, right |
| **Temporal Pole Mid L** | Middle temporal pole, left |
| **Temporal Pole Mid R** | Middle temporal pole, right |
| **Temporal Pole Sup L** | Superior temporal pole, left |
| **Temporal Pole Sup R** | Superior temporal pole, right |
| **Thal IL R** | Thalamus, intralaminar nuclei, right |
| **Thal VA L** | Thalamus, ventral anterior nucleus, left |
| **ThalPuM L** | Thalamus, medial pulvinar, left |
| **Thal IL L** | Thalamus, intralaminar nuclei, left |
| **Thal PuA L** | Pulvinar anterior nucleus of the thalamus, left |
| **Thal VP L** | Thalamus, ventral posterolateral nucleus, left |

a

Left Putamen

right SN

left SN


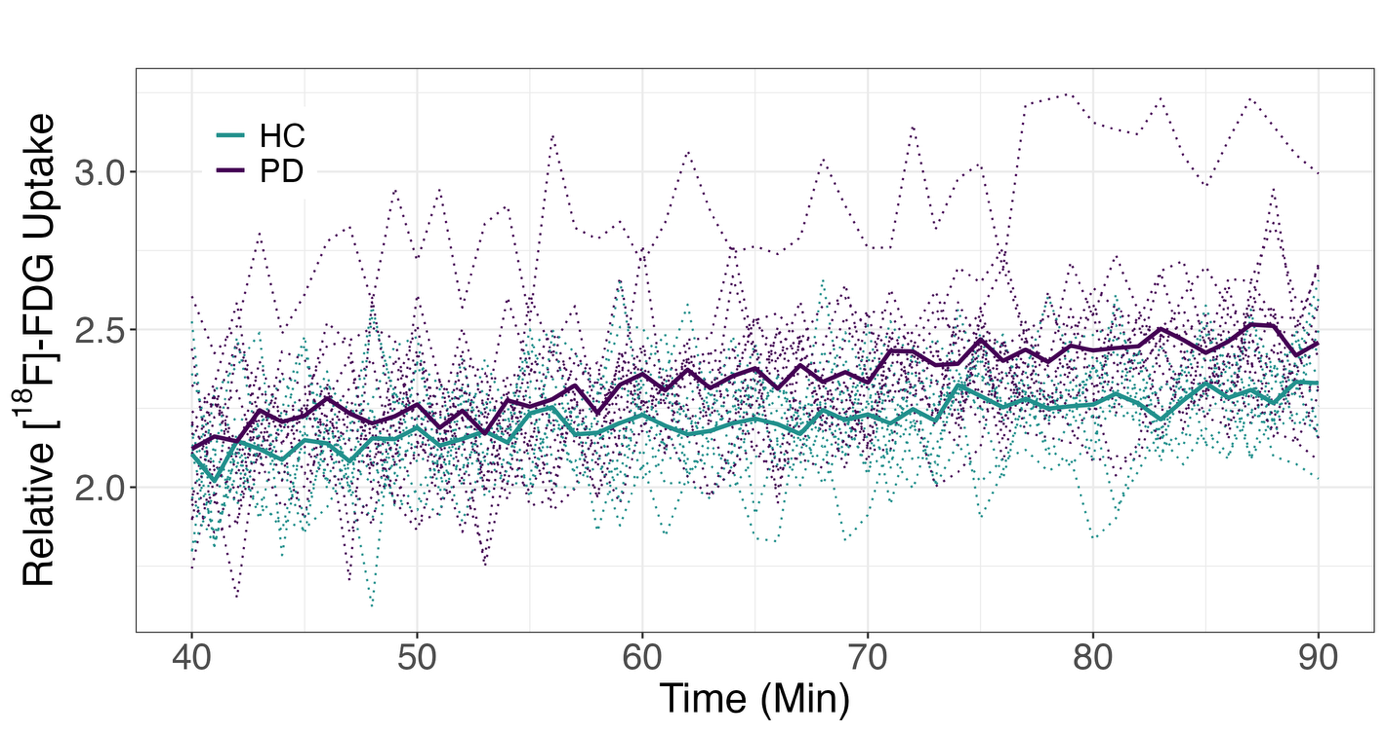

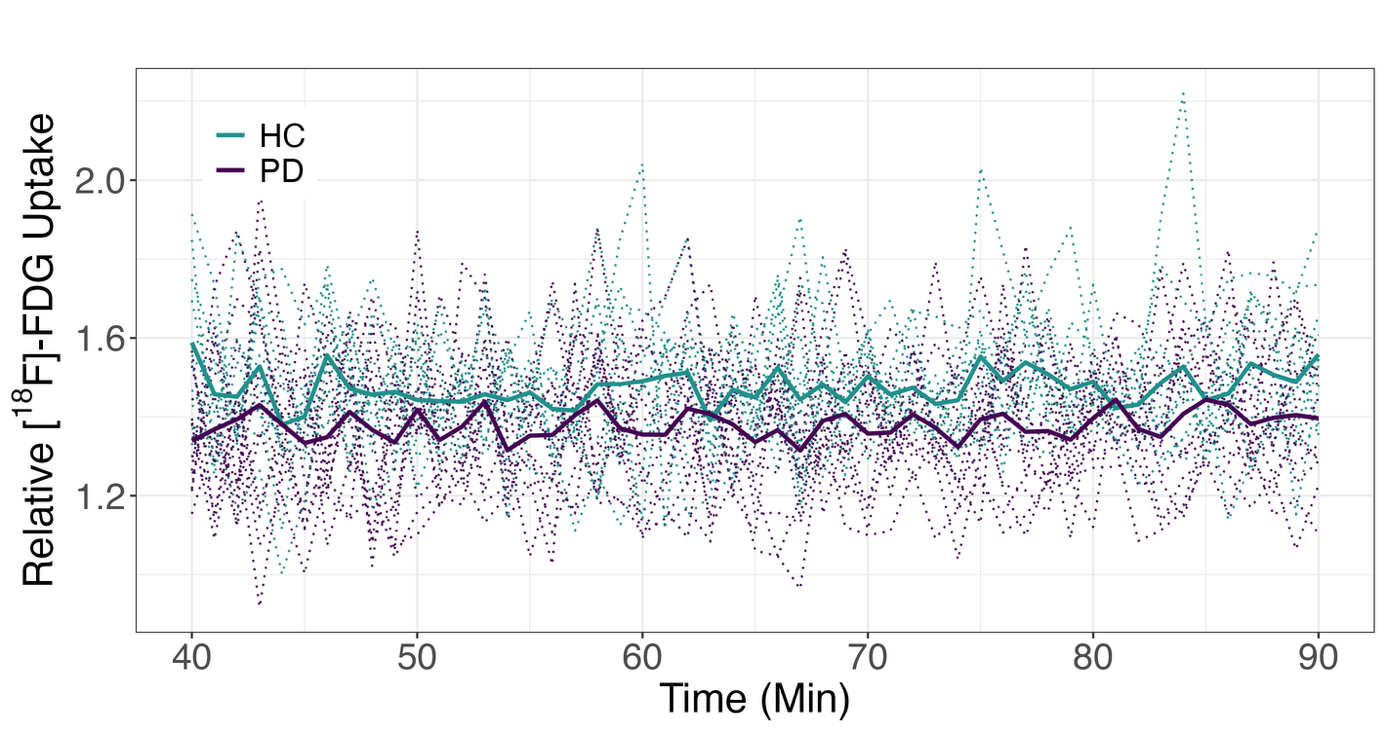

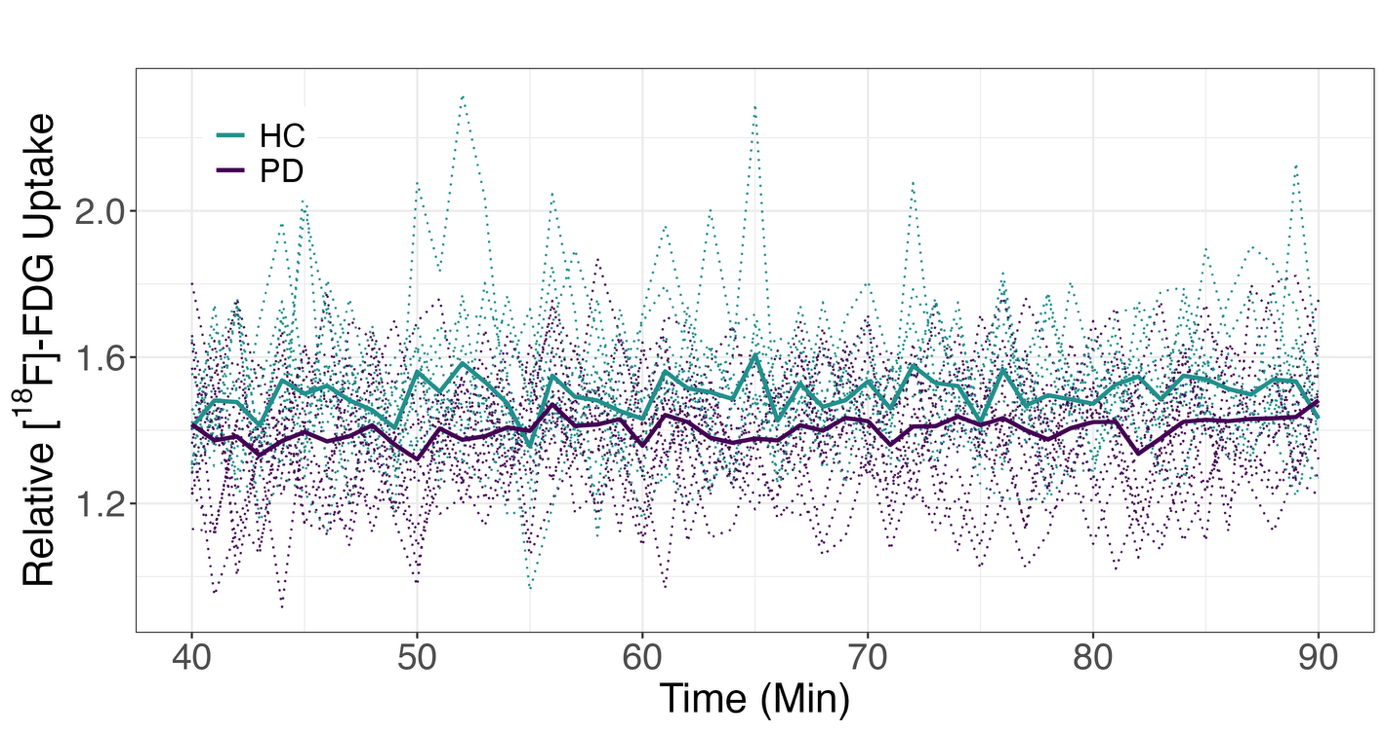


b

c

**Fig. S3:** **Hypometabolic and hypermetabolic clusters in PwPD relative to healthy controls based on metabolic time series comparison.** (a-c) Extracted individual and group mean time courses per cluster of SPM result for the time interval from minute 40 to 90.


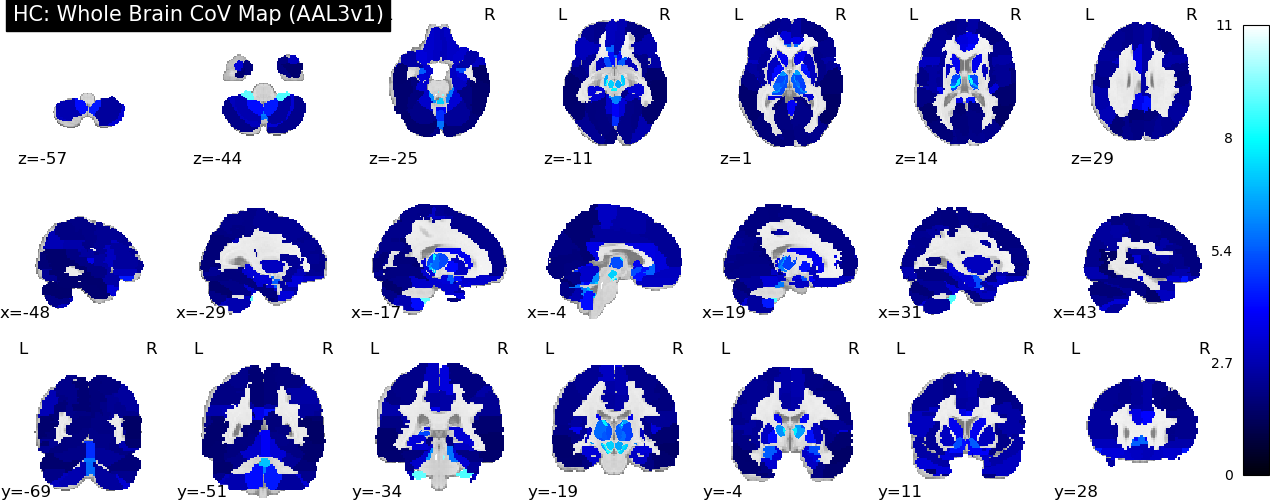

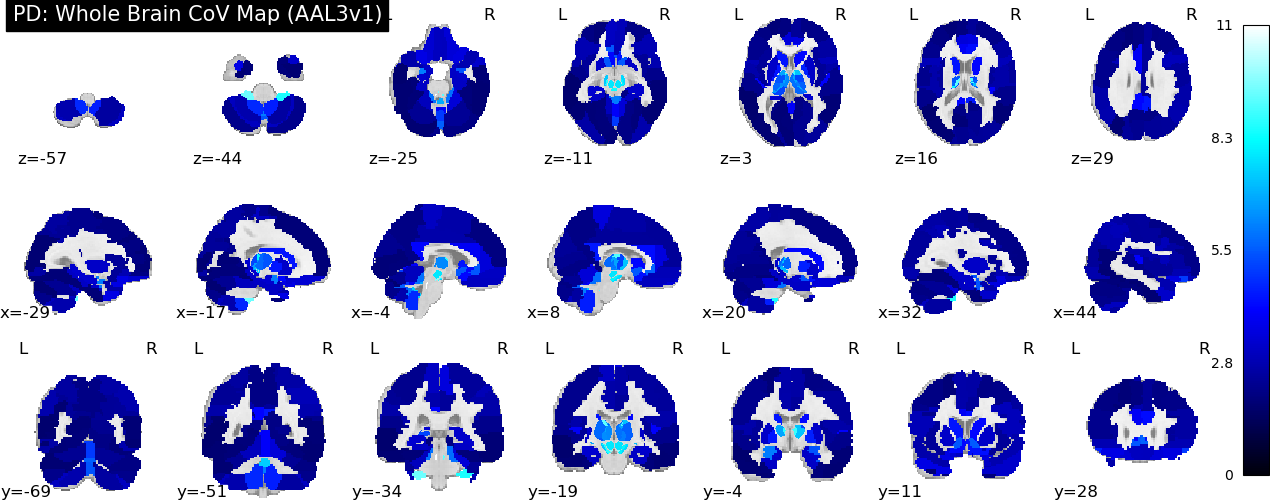


**Fig. S4:** **Whole brain analysis of the coefficient of variation for metabolic time series in controls and PD patients.** Group-level coefficients of variation based on whole brain parcellation with reference to AALv3.

| Region | Temporal signal-to-noise ratio | | | | Test statistic (H) | p-value |
| --- | --- | --- | --- | --- | --- | --- |
|  | HC | PD-MCI | | PD-NC |  |  |
| PCC | 64.2 (55.8; 72.9) | | 51.1 (45.9; 63.6) | 55.2 (49.0; 60.8) | 3.07 | 0.216 |
| SMS | 55.7 (50.1; 85.0) | | 47.8 (40.7; 53.4) | 53.6 (43.4; 66.6) | 3.34 | 0.188 |
| ACC | 32.7 (31.7; 41.6) | | 30.1 (29.9; 31.3) | 32.0 (28.8; 34.8) | 2.56 | 0.279 |
| LPFC-R | 34.3 (26.6; 37.8) | | 25.9 (24.1; 26.5) | 26.6 (24.3; 32.2) | 2.92 | 0.232 |

**Supplementary Table 6: Signal stability in resting-state network nodes on group-level.** Temporal signal-to-noise ratio (tSNR) per group are expressed as median accompanied by 25% and 75% quantiles in brackets. The tSNR values were tested for between-group differences were by using the non-parametric Kruskal-Wallis test.
